# NONO interacts with nuclear PKM2 and directs histone H3 phosphorylation to promote triple-negative breast cancer metastasis

**DOI:** 10.1186/s13046-025-03343-5

**Published:** 2025-03-10

**Authors:** Qixiang Li, Hongfei Ci, Pengpeng Zhao, Dongjun Yang, Yi Zou, Panhai Chen, Dongliang Wu, Wenbing Shangguan, Wenyang Li, Xingjun Meng, Mengying Xing, Yuzhong Chen, Ming Zhang, Bing Chen, Lingdong Kong, Ke Zen, David C. S. Huang, Zhi-Wei Jiang, Quan Zhao

**Affiliations:** 1 The State Key Laboratory of Pharmaceutical Biotechnology, Department of Hematology, The Affiliated Drum Tower Hospital of Nanjing University Medical School, China-Australia Institute of Translational Medicine, School of Life Sciences, Nanjing University, Nanjing, 210023 China; 2 Department of Pathology/ Ophthalmology/Surgical Oncology, The First Affiliated Hospital of Bengbu Medical College, Bengbu, 233000 China; 3 Department of Medicinal Chemistry, School of Pharmacy, China Pharmaceutical University, Nanjing, 211198 China; 4 China-Australia Institute of Translational Medicine Co. Ltd., Nanjing, 211500 China; 5 The Walter and Eliza Hall Institute of Medical Research, Department of Medical Biology, University of Melbourne, Melbourne, VIC 3052 Australia; 6 Department of General Surgery, The Affiliated Hospital of Nanjing University of Chinese Medicine, Nanjing, 210029 China

**Keywords:** Transcription, Epigenomics, H3T11ph, NONO, PKM2, Triple-negative breast cancer, Metastasis

## Abstract

**Background:**

Emerging evidence has revealed that PKM2 has oncogenic functions independent of its canonical pyruvate kinase activity, serving as a protein kinase that regulates gene expression. However, the mechanism by which PKM2, as a histone kinase, regulates the transcription of genes involved in triple-negative breast cancer (TNBC) metastasis remains poorly understood.

**Methods:**

We integrated cellular analysis, including cell viability, proliferation, colony formation, and migration assays; biochemical assays, including protein interaction studies and ChIP; clinical sample analysis; RNA-Seq and CUT&Tag data; and xenograft or mammary-specific gene knockout mouse models, to investigate the epigenetic modulation of TNBC metastasis via NONO-dependent interactions with nuclear PKM2.

**Results:**

We report that the transcription factor NONO directly interacts with nuclear PKM2 and directs PKM2-mediated phosphorylation of histone H3 at threonine 11 (H3T11ph) to promote TNBC metastasis. We show that H3T11ph cooperates with TIP60-mediated acetylation of histone H3 at lysine 27 (H3K27ac) to activate *SERPINE1* expression and to increase the proliferative, migratory, and invasive abilities of TNBC cells in a NONO-dependent manner. Conditional mammary loss of NONO or PKM2 markedly suppressed *SERPINE1* expression and attenuated the malignant progression of spontaneous mammary tumors in mice. Importantly, elevated expression of NONO or PKM2 in TNBC patients is positively correlated with *SERPINE1* expression, enhanced invasiveness, and poor clinical outcomes.

**Conclusion:**

These findings revealed that the NONO-dependent interaction with nuclear PKM2 is key for the epigenetic modulation of TNBC metastasis, suggesting a novel intervention strategy for treating TNBC.

**Supplementary Information:**

The online version contains supplementary material available at 10.1186/s13046-025-03343-5.

## Background

The latest international cancer survey report shows that there are approximately 20 million new cancer cases and 9.7 million cancer deaths worldwide in the year 2022, among which breast cancer ranks second for approximately 2.3 million new cases and fourth for approximately 0.67 million deaths [1]. Triple-negative breast cancer (TNBC) is a type of breast cancer that lacks the expression of estrogen receptor (ER), progesterone receptor (PR), and human epidermal growth factor receptor 2 (HER2) and has high malignancy, easy metastasis, and poor prognosis [2]. Currently, owing to the lack of specific and effective therapeutic targets, treatments for TNBC are limited, resulting in a shorter survival time for patients with TNBC. The mortality rate within five years of diagnosis for TNBC is as high as 40%, seriously threatening the physical and mental health of women [3]. Therefore, there is an urgent need to explore the pathogenesis and therapeutic strategies of TNBC.

Transcriptional dysregulation has been recognized as a hallmark of cancer, where abnormal gene expression leads to tumor initiation and malignant progression [4, 5]. The aberrant interplay between transcription factors and epigenetic regulators is key to cancer pathogenesis [6]. The multifunctional nuclear protein NONO (also known as P54nrb) was initially defined as a non-POU domain-containing octamer-binding protein belonging to the Drosophila behavior/human splicing (DBHS) family [7, 8]. The protein structure of NONO contains RNA and DNA-binding domains, allowing it to participate in important biological processes such as precursor mRNA splicing, transcriptional regulation, nuclear retention of defective RNA, and DNA damage repair [9]. Our recent study revealed that the interaction between the transcription factors NONO and SOX6 synergistically silences γ-globin gene transcription in human erythroid cells [10]. The C-terminus of NONO has a nuclear localization sequence that assists in its distribution mainly in the nucleus and serves as an important component of paraspeckles [11]. However, NONO rarely functions alone and often interacts with other effector proteins to exert its biological effects [12].

In fact, as a binding protein of the TNBC subtype-specific and highly expressed membrane spike protein Moesin (MSN), NONO plays an important role in guiding the localization of MSN to nuclei and subsequent CREB phosphorylation activation to regulate TNBC progression [13]. Additionally, NONO can bind to EGFR in the nucleus to increase its stability and recruit CBP/P300 to enhance the transcriptional activity of EGFR, thereby enhancing nuclear EGFR-mediated tumorigenesis of TNBC [14]. NONO can also bind to IGFBP3 to activate PARP-dependent DNA damage repair, thereby increasing TNBC chemotherapy tolerance [15]. These studies clearly indicate that NONO is crucial for the development of TNBC. However, little is known about whether and how NONO, as a transcription factor, promotes malignant progression or metastasis of TNBC.

Pyruvate kinase (PK), a rate-limiting enzyme in glycolysis, catalyzes the transfer of a phosphate group from phosphoenolpyruvate (PEP) to adenosine diphosphate (ADP) to yield pyruvate and ATP [16]. In the mammalian genome, the PKM1 and PKM2 isoforms are alternatively spliced products of the *PKM* gene by mutually exclusive use of exon 9 or exon 10, respectively. PKM1 is distributed in many normal differentiated tissues, whereas PKM2 is mainly expressed in most proliferating cells, including fetal and cancerous cells [17, 18]. PKM2 has been characterized as a unique biomarker in cancer and has been shown to promote cancer cell proliferation and metastasis by driving the Warburg effect. However, its role in tumorigenesis in different cancers is controversial [17, 18]. Importantly, in addition to its canonical metabolic enzyme function, PKM2 can translocate into the nucleus and function as a protein kinase and transcriptional co-activator [19, 20]. PKM2 phosphorylates histone H3 at threonine 11 (H3T11ph), which is implicated in transcriptional activation [21]. However, the molecular mechanisms by which PKM2 is recruited to participate in gene transcriptional regulation and is precisely enriched across the genome within the nucleus are poorly understood.

In this study, we found that NONO and PKM2 are important regulatory molecules involved in TNBC metastasis. We showed that NONO can specifically recruit PKM2 to coordinate the phosphorylation of threonine 11 of histone H3 (H3T11ph) and the acetylation of lysine 27 of histone H3 (H3K27ac), activating the transcription of multiple key genes, such as *SERPINE1* (serine protease inhibitor family E member 1, encoding the PAI-1 protein) [22, 23], thus promoting the migration and metastasis of TNBC cells. PAI-1 facilitates tumor cell detachment from the matrix and promotes tumor dissemination and metastasis and has been recommended as a promising biomarker for poor prognosis in primary breast cancer patients by the American Society of Clinical Oncology (ASCO) and the European Organization for Research and Treatment of Cancer (EORTC) clinical operation guidelines [22, 23]. Our study revealed that NONO-dependent PKM2 coordinates histone H3 phosphorylation and acetylation to promote gene transcription and metastasis in TNBC cells.

## Methods

### Cell culture

Human embryonic kidney (HEK293T) cells and two human TNBC cell lines (MDA-MB-231 and BT-549) were obtained from Shanghai Institute of Cell Biology, Chinese Academy of Sciences (Shanghai, China). MDA-MB-231 and HEK293T cells were cultured in Dulbecco’s modified Eagle’s medium (DMEM) (Gibco), and BT-549 cells were cultured in RPMI-1640 medium (Gibco) supplemented with 10% FBS (ExCell Bio) and 1% penicillin-streptomycin (100 U/ml, 100 µg/ml; Gibco). Cells were maintained at 37 °C in a humidified incubator with 5% carbon dioxide. Cells were authenticated by short tandem repeat (STR) profiling and were negative for mycoplasma contamination.

### siRNA infection and shRNA lentiviral transduction

The shRNA lentivirus was produced in HEK293T cells by co-transfection with PLKO.1-shRNA, the viral envelope plasmid pMD2. G, and the viral packaging plasmid psPAX2 using Lipofectamine 3000 (Invitrogen) following the manufacturer’s instructions. The scrambled (Scr) sequence was used as the negative control. At 48 h after infection, the viral supernatants were harvested and used to infect cells or stored at -80 °C. To obtain stable cell lines, the cells were selected using 1 µg/mL puromycin (Yeasen, 60209ES100). The Scr and shRNA sequences used were as follows.

Scr: 5’-CCTAAGGTTAAGTCGCCCTCG-3’.

human NONO shRNA-1: 5’-CAGGCGAAGTCTTCATTCATA-3’.

human NONO shRNA-2: 5’-TCCAGAGAAGCTGGTTATAAA-3’;

human PKM2 shRNA-1: 5’-CTACCACTTGCAATTATTTGA-3’.

human PKM2 shRNA-2: 5’-CCACTTGCAATTATTTGAGGA-3’.

Negative control (NC) and specific siRNAs against NONO, PKM2, and TIP60 were synthesized by GenePharma (Suzhou, China). Cells were transiently transfected with 25 nM siRNA using the Lipofectamine 3000 transfection reagent. The siRNA sequences used were as follows:

NC: 5’-UUCUCCGAACGUGUCACGU-3’.

human NONO siRNA-1: 5’-CCUUACAGUUCGAAACCUU-3’;

human NONO siRNA-2: 5’-GGAAGGCACUCAUUGAGAU-3’;

human PKM2 siRNA-1: 5’-CCAUAAUCGUCCUCACCAA-3’.

human PKM2 siRNA-2: 5’-GCCAUAAUCGUCCUCACCA-3’;

human TIP60 siRNA-1: 5’-GGACAGCUCUGAUGGAAUA-3’;

human TIP60 siRNA-2: 5’-GAUCGAGUUCAGCUAUGAA-3’;

To overexpress PAI-1, human *SERPINE1* cDNA was cloned and inserted into the lentiviral vector pLVX-IRES-mCherry at EcoRI and XbaI sites. Human *KAT5* cDNA was cloned and inserted into the pcDNA3.1(+) vector at BamHI and XhoI sites for TIP60 overexpression.

### Cell proliferation, migration and invasion assays

For the CCK-8 assay, cells were plated in 96-well plates at a concentration of 1.5 × 10^3^ cells per well. After the addition of CCK-8 reagent (Vazyme Biotech, A311-01), the cells were incubated for 1.5 h at 37 °C, after which absorbance at a wavelength of 450 nm was detected to construct a growth curve.

For the colony formation assay, cells were seeded in 6-well plates at a density of 1 × 10^3^ cells/well and cultured for 10 days. The cloned cells were fixed with 4% paraformaldehyde (PFA) for 30 min and stained with 0.1% crystal violet solution for 30 min. Finally, colonies were washed with distilled water, dried, and photographed.

For cell migration assays, 3.5 × 10^4^ cells were seeded on the chamber inserts of the Transwell apparatus (Corning, 3422) in serum-free medium, and medium supplemented with 10% FBS was added to the bottom chamber. After 12 h, the uninvaded cells on the upper surface of the Transwell chamber were removed using a moistened cotton swab. The migrated cells on the lower membrane surface were fixed with 4% PFA for 30 min and stained with a 0.1% crystal violet solution for 30 min. Imaging was performed using a fluorescence microscope at ×100 magnification, and images (six fields per membrane) were acquired using ImageJ software. The invasion assay was performed as described for the migration assay, except that the upper chamber was precoated with 50 µl of Matrigel solution.

### Western blot analysis

Total cell proteins were extracted using a cell lysis buffer (Beyotime, P0013) for western blotting and immunoprecipitation. Histone proteins were extracted using a standard protocol for acid extraction of histones from chromatin as previously reported [24]. Proteins were separated using 10% or 15% SDS-PAGE and transferred onto polyvinylidene difluoride (PVDF) membranes (Roche, Basel, Switzerland). The membranes were blocked for 1 h at room temperature in either 5% nonfat milk or BSA (Sigma) and then incubated with primary antibodies overnight at 4 °C. After incubation with secondary antibodies, the protein bands were visualized using enhanced chemiluminescence (Tanon). All the primary antibodies used in this study are listed in Table S4.

### Quantitative real-time PCR analysis (RT‒qPCR)

Total RNA was prepared using TRIzol reagent (Invitrogen) according to the manufacturer’s instructions and assessed using a Nanodrop 2000 spectrophotometer (Thermo Fisher Scientific). Complementary DNAs (cDNAs) was produced using a HiScript II 1st Strand cDNA Synthesis Kit (Vazyme Biotech, R212-01). Quantitative real-time PCR analysis was performed using the AceQ qPCR SYBR Green Master Mix (Vazyme Biotech, R121-02) in a StepOnePlus RT‒PCR system (Thermo Fisher Scientific). GAPDH was used as a loading control. The primers used for qRT-PCR are listed in Table S5.

### BioID2 pull-down and mass spectrometry analysis

BioID2 pull-down experiments were performed as previously described with minor modifications [25]. Briefly, MDA-MB-231 cells transduced with the NONO-BioID2 fusion protein or BioID2-only control were seeded in 10-cm dishes. When the cells reached 80% confluence, the medium was replaced with complete medium containing 50 µM biotin (Sigma, B4501) and the cells were cultured for 16–18 h. The cells were subsequently lysed in lysis buffer (50 mM Tris HCl (pH 7.4), 500 mM NaCl, 0.2% SDS, 1 mM DTT, and 1× protease inhibitors), 0.1 µl of Pierce universal nuclease (Thermo Fisher Scientific, 88701) was added to each sample, and the mixture was incubated for 10 min at room temperature. After sonication and streptavidin affinity purification, the precipitates were washed with a wash buffer (8 M urea in 50 mM Tris, pH 7.4). The expression of NONO-BioID2 fusion protein was identified by western blot analysis using NONO antibody. Finally, the levels of biotinylated proteins were determined by mass spectrometry in collaboration with the BioProfile (Shanghai, China).

### GST pull-down assay

The GST pull-down assay was performed as previously described [26]. Briefly, the pGEX-6P-1 plasmid encoding GST, full-length GST-NONO and fragments, full-length GST-PKM2, fragments, and mutants, as well as the pET28a plasmid encoding His-NONO and His-PKM2, were expressed in *E. coli* BL21 and purified using glutathione S-transferase beads (GenScript, L00206) or nickel-nitrilotriacetic acid beads (GenScript, L00206) according to standard protocols. The His-PKM2 protein was mixed with purified GST, full-length GST-NONO, or fragments for 4 h at 4 °C. Similarly, His-NONO proteins were incubated with GST, GST-PKM2 full-length or fragments or mutants at 4 °C for 4 h. The beads were washed five times, and the results were analyzed by western blotting.

### Immunofluorescence staining

The cells were cultured overnight on glass coverslips, fixed with 4% PFA for 20 min, and permeabilized with 0.2% Triton X-100 for 20 min. After blocking with 5% BSA for 30 min, cells were incubated with primary antibodies against NONO (HUABIO, ET7108-81) and PKM2 (Proteintech, 60268-1-lg) overnight at 4 °C, followed by incubation with fluorescence-conjugated secondary antibodies for 1 h at room temperature. The cells were then gently washed with PBS and the nuclei were stained with DAPI for 5 min at room temperature. Images were acquired using a confocal laser scanning microscope (Olympus).

### RNA-seq and data analysis

Total RNA was isolated from the NC, NONO-knockdown, and PKM2-knockdown MDA-MB-231 cells. RNA purification, reverse transcription, library construction, and sequencing were performed in collaboration with Majorbio Biotechnology (Shanghai, China) according to the manufacturer’s instructions (Illumina, San Diego, CA, USA). To identify differentially expressed genes (DEGs), gene expression was calculated as transcripts per million reads (TPM). A fold change > 2.0, and a *P* value < 0.05 were used as the cutoff values and were considered to indicate significantly differentially expressed genes.

### Chromatin Immunoprecipitation (ChIP) assay

ChIP assays were performed using MDA-MB-231 cells in accordance with previously described methods [26]. Normal rabbit IgG (ab172730; Abcam) was used as the control. ChIP DNAs was analyzed by quantitative PCR using Rotor-Gene 6000 (Corbett Research). Primer sequences used for ChIP are listed in Table S6.

### CUT&Tag

CUT&Tag was processed according to a previously reported protocol [27]. This experiment was performed in MDA-MB-231 cells, and antibodies against NONO (Abcam, ab70335), H3T11ph (Active motif, 39151), and H3K27ac (Abcam, ab4729) were used in this study. DNA libraries were sequenced on an Illumina NovaSeq 6000 platform (DIATRE Biotechnology, Shanghai, China).

### Tissue microarrays and IHC staining

Human TNBC tissue microarrays (no. TNBC1202) containing a total of 60 pairs of TNBC tissue samples and matched adjacent normal tissues with follow-up clinicopathological data were obtained from Shanghai Superbiotek Co., Ltd. (Shanghai, China). IHC staining was performed according to the standard protocol of the Cell Signaling Technology. Tissue slides were incubated with primary antibodies specific for NONO (HUABIO, ET7108-81), PKM2 (Proteintech, 15822-1-AP), PAI-1 (Abcam, ab125687), and Ki-67 (Abcam, ab15580), followed by incubation with horseradish peroxidase (HRP)-conjugated secondary antibodies. The staining of NONO, PKM2, and PAI-1 in human TNBC tissues was independently assessed using a semi-quantitative H-score system by two experienced pathologists blinded to the clinical data [28].

### Animal studies

Mice were maintained in a specific pathogen-free (SPF) facility on a 12 h light/dark cycle at a controlled temperature (20–23 °C) and humidity (45–65%). The sample size was chosen with adequate power based on the literature and our previous experience [28]. For each experiment, it is indicated in the figure legend. Prior to the experiment, the mice were randomly assigned to different treatment groups. MMTV-PyMT mice on the FVB background were purchased from GemPharmatech (Nanjing, China). For NONO or PKM2 knockdown in MMTV-PyMT (FVB) mice, pAAV-U6-EGFP plasmids were transformed with shRNAs and AAVs were obtained from Syngentech (Beijing, China). AAVs were intraductally injected into the mammary glands of 5-week-old female MMTV-PyMT (FVB background) mice as previously described [29]. After 2 weeks, tumor size was determined once a week, and tumor volumes were calculated using the following formula: volume (mm^3^) = 1/2 × length × width^2^. All mice were euthanized at 12 weeks of age, and tumor and lung samples were collected, weighed, photographed, and stained with H&E. The Scr sequence was used as the negative control. The shRNA sequences used were as follows:

Scr: 5’-CCTAAGGTTAAGTCGCCCTCGC-3’.

Mouse NONO shRNA-1: 5’-GCTGCAACAATGGAAGGAATT-3’.

Mouse NONO shRNA-2: 5’-ACACGAACCCTAGCGGAAATT-3’.

Mouse PKM2 shRNA-1: 5’-CTACCACTTGCAGCTATTCGA-3’.

Mouse PKM2 shRNA-1: 5’-CCACTTGCAGCTATTCGAGGA-3’.

For xenograft models, 6-week-old female BALB/c nude mice were purchased from GemPharmatech (Nanjing, China). MDA-MB-231 cells were infected individually with Scr, NONO-KD, or NONO-KD + PAI-1 lentivirus to establish stable cell lines. Subsequently, each mouse was injected subcutaneously with 5 × 10^6^ cells (suspended in 100 µl of PBS with 100 µl of Matrigel). Tumor size was measured every 3 days with a caliper, and the average tumor volume reached 100 mm^3^. After 21 days, all nude mice were sacrificed, and subcutaneous tumors were harvested, weighed, and photographed.

To establish mammary-specific NONO or PKM2 knockout models in spontaneous mammary tumor mice, MMTV-Cre and NONO-floxed mice on a C57BL/6 strain background were obtained from GemPharmatech (Nanjing, China). MMTV-PyMT mice and PKM2-floxed mice on a C57BL/6 background were purchased from Jackson Laboratory (New York, USA). The overall tumor burden was measured once a week using a digital caliper beginning at week 16, and the mice were humanely euthanized at 24 weeks of age. Mammary tumors were harvested for histology, flash-frozen for protein and RNA isolation, and subjected to IHC staining with the indicated antibodies, whereas the lungs were extracted for H&E staining. A blinding strategy was used whenever possible when assessing outcomes. The sequences of the PCR primers used for genotyping transgenic mice are listed in Table S7.

### Statistical analysis

All data are presented as mean ± SD unless otherwise indicated. GraphPad Prism software (version 7.0) was used to assess significant differences between groups. Comparisons between two groups were performed using an unpaired two-tailed Student’s t-test. Multiple comparisons were performed using a one-way analysis of variance (ANOVA). *P* < 0.05. One, two, three, and four asterisks indicate *P* < 0.05, *P* < 0.01, *P* < 0.001, and *P* < 0.0001, respectively.

## Results

### NONO directly interacts with nuclear PKM2 in TNBC cells

**Fig. 1:**
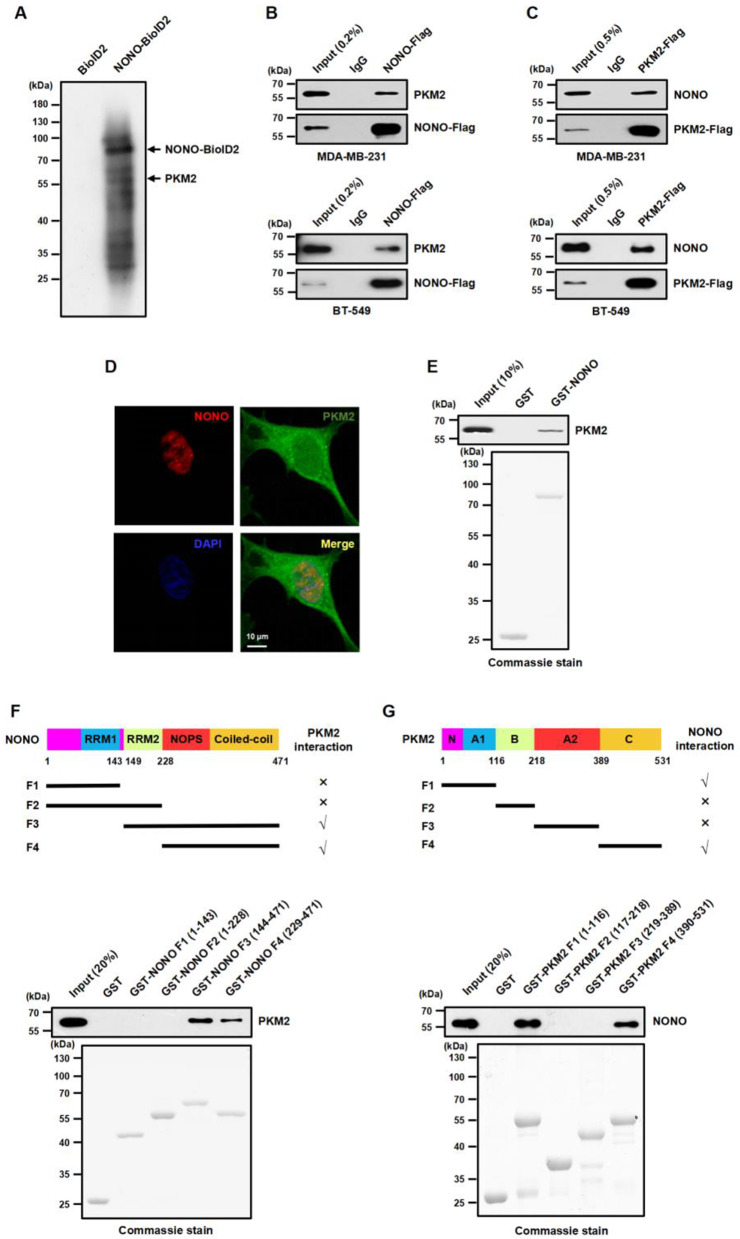
NONO directly interacts with nuclear PKM2. **(A)** Proteins biotinylated by BioID2 alone or NONO-BioID2 in MDA-MB-231 cells were examined via western blotting with HRP-conjugated streptavidin following SDS‒PAGE separation. PKM2 was identified via quantitative mass spectrometry and indicated based on its molecular weight. **(B)** Endogenous PKM2 was coimmunoprecipitated with anti-Flag M2 Sepharose beads in MDA-MB-231 and BT-549 cells overexpressing Flag-tagged NONO. IgG served as the negative control. **(C)** Coimmunoprecipitation of endogenous NONO with PKM2-Flag from MDA-MB-231 and BT-549 cells. **(D)** The cellular localization of NONO and PKM2 in MDA-MB-231 cells was examined by immunofluorescence with anti-NONO and anti-PKM2 antibodies and by nuclear counterstaining with DAPI. **(E)** A GST pull-down assay was used to detect the interaction between NONO and PKM2 in vitro (top). Purified GST and GST-NONO fusion proteins preabsorbed to glutathione-Sepharose beads were incubated with the prokaryotic His-PKM2 fusion protein. GST and GST-NONO fusion proteins were visualized by Coomassie blue staining (bottom). **(F)** Schematic diagram of NONO truncation mutants (top). The binding of His-PKM2 fusion proteins to purified GST, GST-NONO fragments F1 (1-143 aa), F2 (1-228 aa), F3 (144–471 aa), and F4 (229–471 aa) was assessed by a GST pull-down assay (middle). GST, GST-NONO F1, F2, F3, and F4 fusion proteins were visualized by Coomassie blue staining (bottom). **(G)** Schematic diagram of PKM2 in different truncated constructs (top). The binding of the His-NONO fusion protein to purified GST, GST-PKM2 fragments F1 (1-116 aa), F2 (117–218 aa), F3 (219–389 aa), and F4 (390–531 aa) was assessed by a GST pull-down assay (middle). GST, GST-PKM2 F1, F2, F3, and F4 fusion proteins were visualized by Coomassie blue staining (bottom)

The paraspeckle protein NONO plays a critical role in TNBC, although its direct downstream transcriptional target genes are unknown [13–15]. To identify potential proteins that interact with NONO, we used a proximity-dependent biotinylation identification approach known as BioID2 [25], in which NONO is fused to the N-terminus of the BioID2 vector and transduced into human MDA-MB-231 cells. An empty vector containing only BirA biotin ligase was used as the control. After biotin-streptavidin affinity purification, proteins associated with NONO were analyzed using quantitative mass spectrometry. Among the proteins that were specifically enriched in NONO-BioID2-expressing cells, PKM2 was identified as an NONO-interacting protein in the top 10 list (Fig. 1A, Table S1). In addition, we found that SFPQ, PSPC1, and MSN [12–14], which are NONO-binding proteins, are also present. To confirm the interaction between NONO and PKM2, we performed co-immunoprecipitation (co-IP) experiments and found that NONO interacted with PKM2 in both MDA-MB-231 and BT-549 cells (Fig. 1B and C). Furthermore, immunofluorescence staining revealed that NONO co-localized with PKM2 in the nuclei of MDA-MB-231 cells (Fig. 1D). These results agree with previous observations that PKM2 is present in the nucleus [20, 21, 30].

Next, we found that NONO directly interacted with PKM2 in the GST pull-down experiments (Fig. 1E). To further characterize the determinants mediating the association between NONO and PKM2, we mapped NONO protein domains using GST pull-down experiments and demonstrated that the NOPS and coiled-coil domains of NONO were sufficient for their interaction (Fig. 1F). Similarly, we demonstrated that the A and C domains of PKM2 were responsible for its interaction with NONO (Fig. 1G), indicating that NONO interacts with PKM2 through multiple domains. Taken together, these data indicate that PKM2 is a novel protein that interacts with NONO in the TNBC cell nuclei.

### NONO expression is associated with the prognosis of TNBC patients, and NONO is required for TNBC cell metastasis

**Fig. 2:**
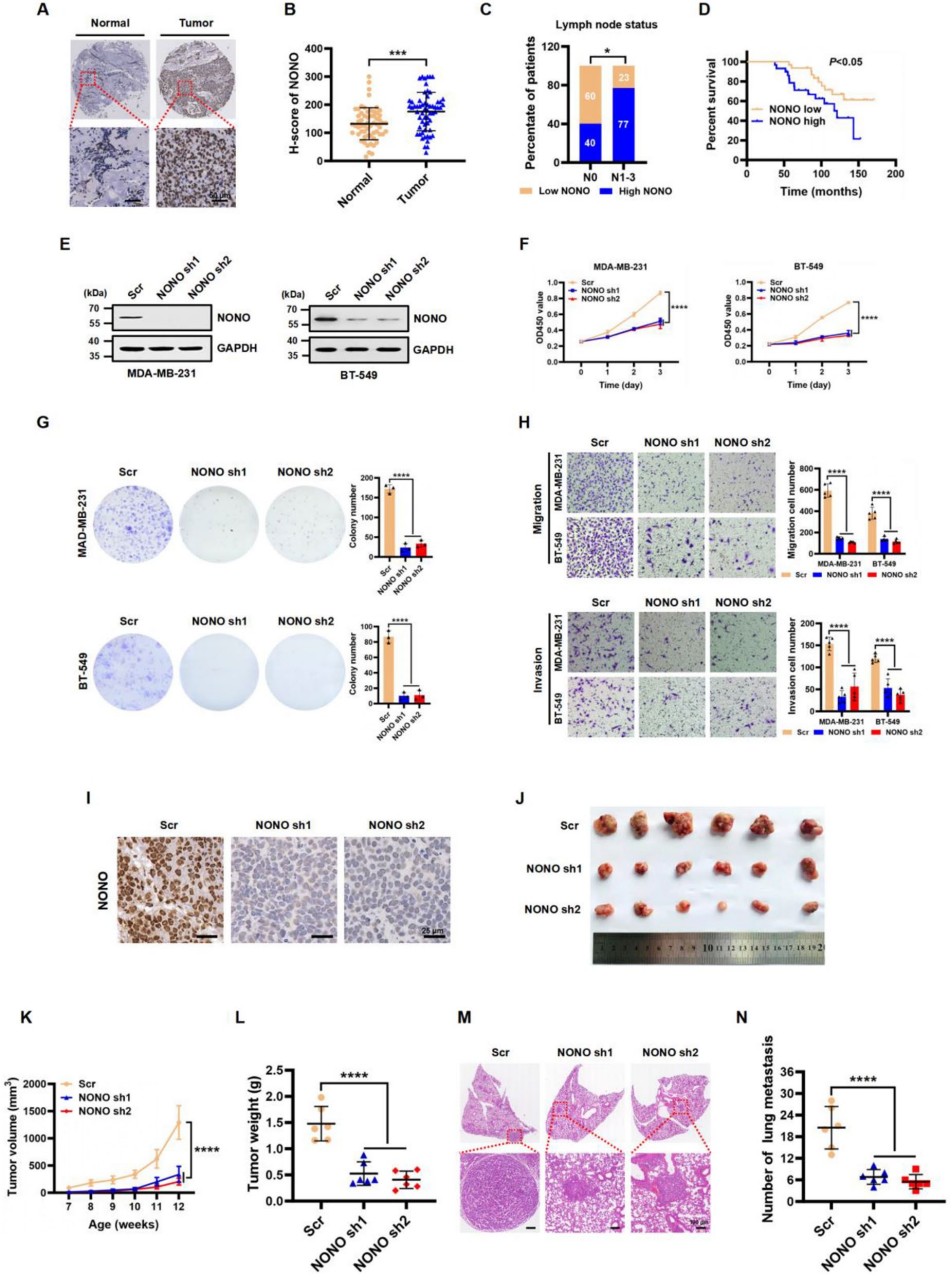
Upregulation of NONO is associated with poor prognosis in TNBC patients and promotes TNBC cell proliferation and metastasis in vitro and in vivo. **(A)** Representative images of IHC staining of NONO in matched normal tissues (*n* = 60) and TNBC tissues (*n* = 60). Scale bar, 50 μm. **(B)** Quantitative analysis of NONO expression levels in matched normal tissue (Normal) and TNBC tissue (Tumor) samples. The data are presented as the mean ± SD. ***P < 0.001. **(C)** Percentage of TNBC patients with high expression or low expression of NONO stratified according to lymph node status (N0 or N1–3) (*n* = 60); two-sided Fisher’s exact test, *P < 0.05. **(D)** Kaplan‒Meier plot of the overall survival of TNBC patients with high expression (*n* = 31) or low expression (*n* = 29) of NONO. Long-rank test, *P* < 0.05. **(E)** NONO protein expression was detected by western blot in MDA-MB-231 and BT-549 cells infected with scramble control (Scr) or NONO shRNA lentivirus. GAPDH was used as a loading control. **(F)** Cell proliferation was examined by CCK-8 assays in MDA-MB-231 and BT-549 cells infected with Scr, NONO sh1, or NONO sh2 lentivirus. The data are presented as the mean ± SD. ****P < 0.0001 compared to the Scr control group. **(G)** Colony formation assay of MDA-MB-231 and BT-549 cells with Scr control or NONO knockdown. The number of colonies is shown in the bar graph (right panels). The data are presented as the mean ± SD (*n* = 3). ****P < 0.0001 compared to the Scr control group. **(H)** Representative images of the migration (top panels) and invasion ( bottom panels) of MDA-MB-231 and BT-549 cells in the Scr control and NONO knockdown groups. The data are presented as the mean ± SD (*n* = 5). ****P < 0.0001 compared to the Scr control group. **(I)** Representative IHC staining of NONO in mammary tumor tissues from MMTV-PyMT (FVB background) mice injected with Scr, NONO sh1, and NONO sh2 adeno-associated viruses (AAVs). Scale bar, 25 μm. **J-L.** Representative images of excised tumors (**J**), tumor growth curves (**K**), and tumor weights (**L**); representative H&E staining images of lungs (**M**); and images of the number of lung metastases (**N**) in MMTV-PyMT (FVB background) mice injected with Scr, NONO sh1, and NONO sh2 AAVs. Scale bar, 100 μm. All data are presented as the mean ± SD (*n* = 6). ****P < 0.0001 compared to the Scr control group

To investigate the clinical significance of NONO expression in patients with TNBC, we first analyzed NONO expression by immunohistochemistry (IHC) using tissue microarrays containing 60 TNBC samples and matched adjacent normal tissues. We observed that NONO expression was significantly upregulated in TNBC tissues compared to that in the adjacent normal tissues (Fig. 2A and B). Notably, NONO expression correlated with tumor size and higher-grade lymph node status in patients with TNBC (Fig. 2C; Table S2). Importantly, Kaplan-Meier survival analysis revealed that TNBC patients with high NONO expression had a shorter overall survival (Fig. 2D). The expression levels of NONO in TNBC tissues were significantly higher than those in non-TNBC tissues (Additional file 1: Fig. S1A). These results indicate that NONO is upregulated in human TNBC tissues and correlates with poor prognosis in patients with TNBC, suggesting that NONO may promote cancer cell invasion during malignant progression.

To determine the effect of NONO on cell growth and invasion, two independent short hairpin RNAs (shRNAs) targeting NONO were used to silence NONO expression. The knockdown efficiency of NONO in human MDA-MB-231 and BT-549 TNBC cell lines was determined by western blot analysis (Fig. 2E). CCK-8 and colony formation assays indicated that the proliferation of MDA-MB-231 and BT-549 cells was significantly inhibited upon NONO knockdown compared with that of scrambled control (Scr) cells (Fig. 2F and G). Transwell assays revealed that the migratory and invasive abilities of TNBC cells were significantly lower in the NONO knockdown group than in the Scr group (Fig. 2H). In addition, the well-established MMTV-PyMT (FVB background) transgenic murine model of spontaneous mammary tumors [31] was used to determine whether NONO directly regulated tumorigenesis. Adeno-associated viruses (AAVs) were used to achieve NONO knockdown in mouse breast tissue. The efficiency of NONO knockdown in mouse breast cancer tissues was subsequently evaluated using immunohistochemical (IHC) analysis. (Fig. 2I). Remarkably, tumor growth was significantly delayed upon NONO knockdown compared with that of Scr control group (Fig. 2J, K, and L). More importantly, NONO knockdown substantially reduced the number of metastatic lung nodules (Fig. 2M and N). These results indicated that NONO is crucial for TNBC growth and metastasis.

### PKM2 expression is upregulated in TNBC, and knockdown of PKM2 inhibits TNBC cell metastasis

**Fig. 3:**
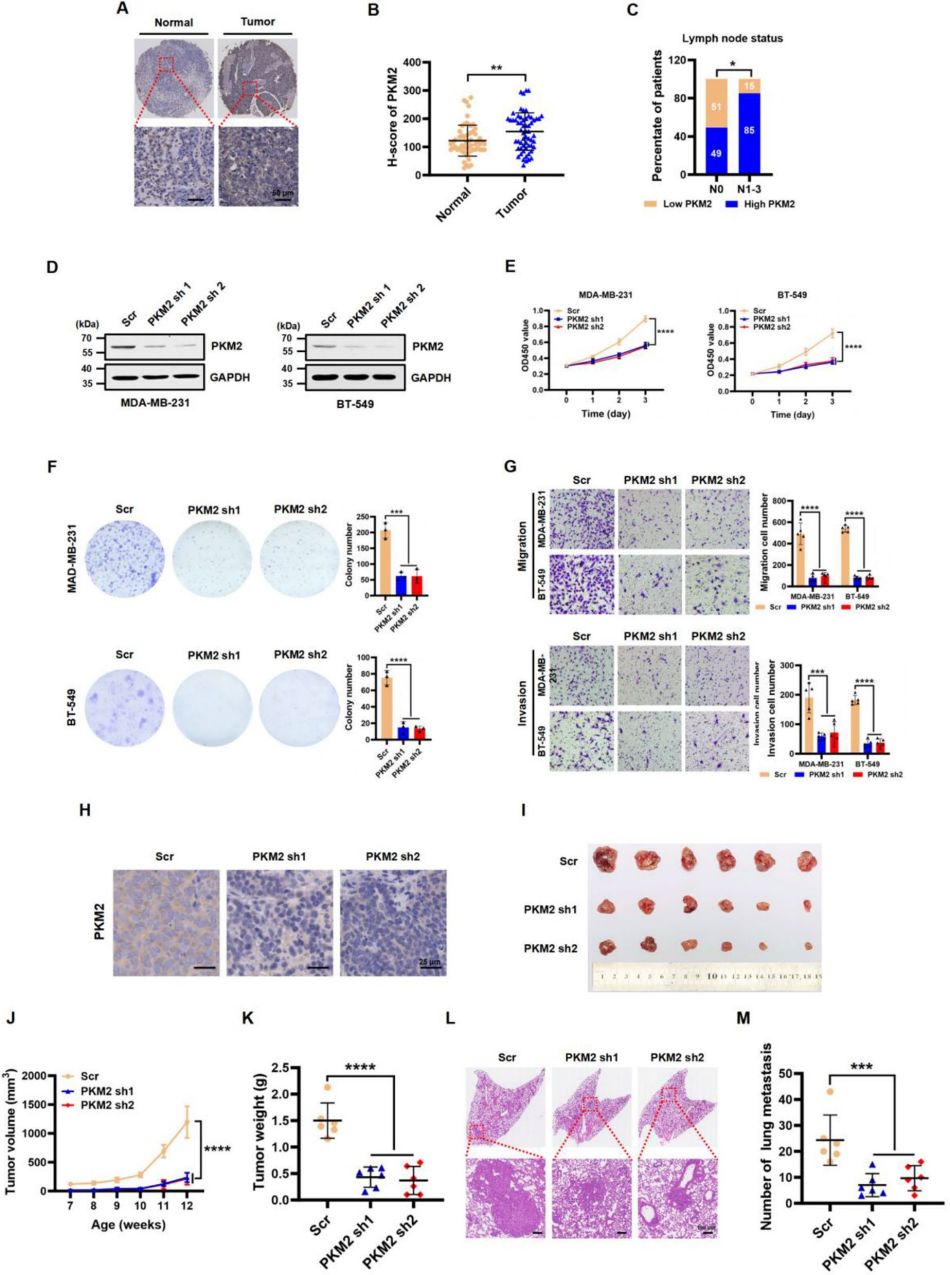
PKM2 expression is upregulated in TNBC, and knockdown of PKM2 diminishes TNBC cell proliferation and metastasis both in vitro and in vivo. **(A)** Representative images of IHC staining of PKM2 in matched normal tissues (*n* = 60) and TNBC tissues (*n* = 60). Scale bar, 50 μm. **(B)** Quantitative analysis of PKM2 expression levels in matched normal tissue (Normal) and TNBC tissue (Tumor) samples. The data are presented as the mean ± SD. **P < 0.01. **(C)** Percentage of TNBC patients with high expression or low expression of PKM2 stratified according to lymph node status (N0 or N1–3) (*n* = 60); two-sided Fisher’s exact test, *P < 0.05. **(D)** PKM2 protein expression was detected by western blot in MDA-MB-231 and BT-549 cells infected with the Scr, PKM2 sh1, or sh2 lentivirus. GAPDH served as a loading control. **(E)** Cell proliferation was examined by CCK-8 assays in MDA-MB-231 and BT-549 cells infected with the Scr, PKM2 sh1, or sh2 lentivirus. The data are presented as the mean ± SD. ****P < 0.0001 compared to the Scr control group. **(F)** Colony formation assay of MDA-MB-231 and BT-549 cells with Scr control or PKM2 knockdown. The number of colonies is shown in the bar graph (right panels). The data are presented as the mean ± SD (*n* = 3). ***P < 0.001, ****P < 0.0001 compared to the Scr control group. **(G)** Representative images of the migration (top panels) and invasion (bottom panels) of MDA-MB-231 and BT-549 cells after Scr control or PKM2 knockdown. The data are presented as the mean ± SD (*n* = 5). ***P < 0.001, ****P < 0.0001 compared to the Scr control group. **(H)** Representative IHC staining of PKM2 in mammary tumor tissues from MMTV-PyMT (FVB background) mice injected with Scr, PKM2 sh1, and PKM2 sh2 AAVs. Scale bar, 25 μm. **I-K** Representative images of excised tumors (**I**), tumor growth curves (**J**), and tumor weights (**K**); representative H&E staining images of lungs (**L**); and the number of lung metastases (**M**) from MMTV-PyMT (FVB background) mice injected with Scr, PKM2 sh1, or sh2 AAVs. Scale bar, 100 μm. All data are presented as the mean ± SD (*n* = 6). ***P < 0.001, ****P < 0.0001 compared to the Scr control group

Similarly, to investigate the functional role of PKM2 in TNBC progression, we examined the clinical significance of PKM2 expression in TNBC specimens and adjacent normal tissues. IHC analysis revealed that PKM2 expression was significantly higher in human TNBC tissues compared to adjacent normal tissues. Importantly, PKM2 expression was associated with larger tumor size and higher-grade lymph node status in TNBC patients. (Fig. 3A, B, C, and Table S3). Additionally, PKM2 expression in TNBC tissues was significantly higher than that in non-TNBC tissues. (Additional file 1: Fig. S1B), which is consistent with previous results [30]. and further suggests that PKM2 may promote cancer cell invasion during malignant progression.

Furthermore, we investigated whether PKM2 plays an oncogenic role in human TNBC cells. We stably transduced PKM2-targeting shRNAs into two TNBC cell lines, MDA-MB-231 and BT-549, and confirmed the knockdown efficiency by Western blot (Fig. 3D). Silencing PKM2 expression significantly impeded cell proliferation and weakened colony formation ability compared to the Scr control group (Fig. 3E and F). Additionally, PKM2 knockdown suppressed cell migration and invasion in both MDA-MB-231 and BT-549 cells compared to the Scr group (Fig. 3G). To further explore the in vivo effects of PKM2 on mammary tumor development, we used AAV-mediated knockdown of PKM2 in the MMTV-PyMT mouse breast cancer model (FVB background). We found that PKM2 downregulation significantly impaired tumor growth compared to the Scr group (Fig. 3H, I, J and K). Notably, PKM2 depletion also inhibited lung metastatic ability and significantly reduced the formation of large metastatic nodules (Fig. 3L and M). Therefore, our in vitro knockdown experiments and in vivo animal study results indicated that PKM2 is crucial for TNBC growth and metastasis.

### *SERPINE1* is a key transcriptional target of NONO and PKM2 in TNBC cells

**Fig. 4:**
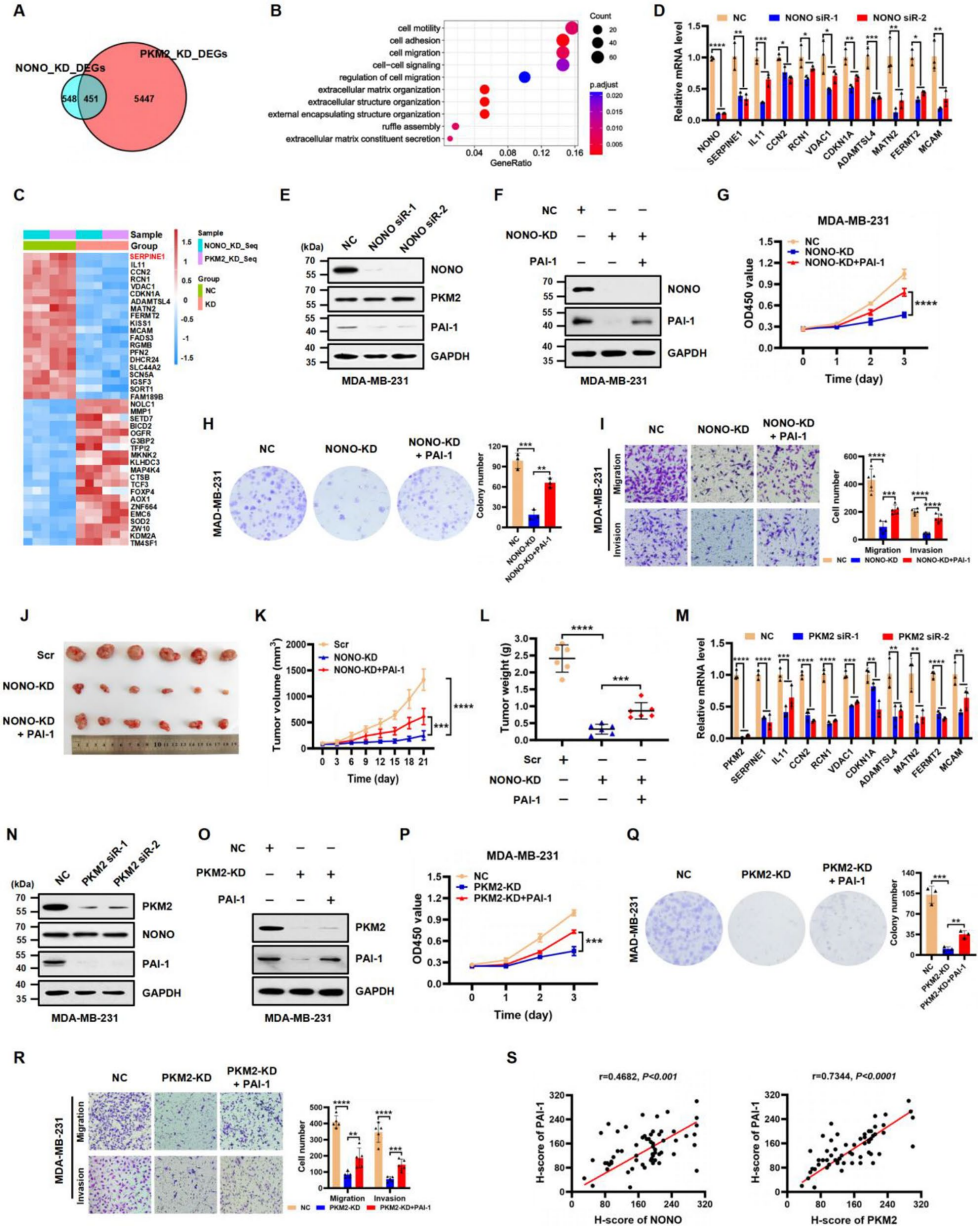
*SERPINE1* is a key transcriptional target of NONO and PKM2 in TNBC. **(A)** Venn diagram depicting the overlap of NONO- and PKM2-regulated differentially expressed genes (DEGs) in MDA-MB-231 cells obtained from RNA-seq analysis. **(B)** Gene ontology (GO) analysis to show the top 10 pathways with the smallest p.adjust values, pathway shown as the ordinate and gene ratio shown as the abscissa. The size indicated the number; the redder the color, the smaller the p.adjust value. **(C)** Heatmap showing the top 20 most differentially expressed genes whose expression was coupregulated or codownregulated by NONO or PKM2. NC: negative control; KD: knockdown. **(D)** Relative mRNA levels of *SERPINE1*, *IL11*, *CCN2*, *RCN1*, *VDAC1*, *CDKN1A*, *ADAMTSL4*, *MATN2*, *FERMT2* and *MCAM* normalized to those of *GAPDH* were examined by RT‒qPCR in negative control (NC) or NONO-silenced MDA-MB-231 cells. The data are presented as the mean ± SD (*n* = 3). *P < 0.05, **P < 0.01, ***P < 0.001, ****P < 0.0001 compared to NC. **(E)** The protein expression of PAI-1 in NC and NONO-KD MDA-MB-231 cells was assessed by western blot analyses with the indicated antibodies. **(F)** Western blot analysis of the indicated proteins in MDA-MB-231 cells treated with NC, NONO-KD, or NONO-KD + PAI-1. GAPDH served as a loading control. **(G)** Cell proliferation was examined by a CCK-8 assay in MDA-MB-231 cells treated with NC, NONO-KD, or NONO-KD + PAI-1. The data are presented as the mean ± SD (*n* = 5). ****P < 0.0001 compared to the NONO-KD group. **(H)** Colony formation was determined in MDA-MB-231 cells treated with NC, NONO-KD, or NONO-KD + PAI-1. The data are presented as the mean ± SD (*n* = 3). **P < 0.01, ***P < 0.001 compared to the corresponding control. **(I)** Representative images of the migration (top panels) and invasion (bottom panels) of MDA-MB-231 cells treated with NC, NONO-KD, or NONO-KD + PAI-1. The data are presented as the mean ± SD (*n* = 5). ***P < 0.001, ****P < 0.0001 compared to the corresponding control. **J-L.** Photographs (**J**), tumor growth curves (**K**), and tumor weights (**L**) of MDA-MB-231 xenograft tumors from the scramble (Scr), NONO-KD, and NONO-KD + PAI-1 groups. The data are presented as the mean ± SD (*n* = 6). ***P < 0.001, ****P < 0.0001 compared to the corresponding control. **M.** Relative mRNA levels of *SERPINE1*, *IL11*, *CCN2*, *RCN1*, *VDAC1*, *CDKN1A*, *ADAMTSL4*, *MATN2*, *FERMT2* and *MCAM* (normalized to *GAPDH*) were examined by RT‒qPCR in NC and PKM2-KD MDA-MB-231 cells. The data are presented as the mean ± SD (*n* = 3). **P < 0.01, ***P < 0.001, ****P < 0.0001 compared to NC. **N.** The protein expression of PAI-1 in NC and PKM2-KD MDA-MB-231 cells was assessed by western blot analyses with the indicated antibodies. **O.** Western blot analysis of the indicated proteins in MDA-MB-231 cells treated with NC, PKM2-KD, or PKM2-KD + PAI-1. GAPDH served as a loading control. **P.** Cell proliferation was examined by a CCK-8 assay in MDA-MB-231 cells treated with NC, PKM2-KD, or PKM2-KD + PAI-1. The data are presented as the mean ± SD (*n* = 5). ***P < 0.001 compared to the PKM2-KD group. **Q.** Colony formation was determined in MDA-MB-231 cells treated with NC, PKM2-KD, or PKM2-KD + PAI-1. The data are presented as the mean ± SD (*n* = 3). **P < 0.01, ***P < 0.001 compared with the PKM2-KD group. **R.** Representative images of the migration (top panels) and invasion (bottom panels) of MDA-MB-231 cells treated with NC, PKM2-KD, or PKM2-KD + PAI-1. The data are presented as the mean ± SD (*n* = 5). **P < 0.01, ****P < 0.0001 compared to the corresponding control. **S.** Pearson correlation scatter plot of H scores for NONO, PKM2, and PAI-1 in human TNBC tissues (*n* = 60)

Next, we sought to understand how NONO and PKM2 regulate the migration and invasion of TNBC cells. To identify potential transcriptional targets of the NONO/PKM2 complex, we performed RNA-seq analysis to profile co-regulated target genes. Through integrated analysis of RNA-seq results following NONO and PKM2 knockdown in MDA-MB-231 cells, we found that nearly half of the differentially expressed genes DEGs (451/999) following NONO knockdown were also regulated by PKM2 (Fig. 4A, GSE266177). Importantly, bioinformatic analysis revealed that these genes are involved in modulating cell motility, adhesion, migration, and other biological processes (Fig. 4B). Further analysis revealed that *SERPINE1* (encoding the PAI-1 protein) [22, 23, 32] was the gene whose expression was most downregulated by either NONO or PKM2 knockdown (Fig. 4C, GSE266177). In the top 10 heatmaps, IL11, CCN2, RCN1, VDAC1, CDKN1A (P21), ADAMTSL4, MATN2, FERMT2, and MCAM, which play key roles in cell growth, adhesion, migration, and differentiation [33–40], were significantly downregulated when either NONO or PKM2 was knocked down. Given that PAI-1 facilitates tumor cell detachment from the matrix and promotes tumor dissemination and metastasis, which has been recommended as a promising biomarker for poor prognosis in primary breast cancer patients by the American Society of Clinical Oncology (ASCO) and the European Organization for Research and Treatment of Cancer (EORTC) clinical operation guidelines [22, 23], we selected *SERPINE1* as a primary target for further study. We confirmed that NONO knockdown in MDA-MB-231 cells significantly reduced the expression of *SERPINE1* at both the transcriptional and protein level (Fig. 4D and E). Subsequently, we examined whether the enforced expression of PAI-1 would compensate for NONO knockdown. We first overexpressed exogenous PAI-1 in NONO-depleted MAD-MB-231 cells (Fig. 4F). We then performed CCK-8, colony formation, and Transwell assays using these cells. We found that the overexpression of PAI-1 largely rescued the reduced proliferation, migration, and invasion in NONO-depleted cells (Fig. 4G, H, and I). To further support the in vitro results, we performed in vivo analysis using xenograft models in nude mice. We showed that overexpression of PAI-1 partially reversed the growth defects in NONO-depleted MDA-MB-231 xenograft tumors (Fig. 4J, K, and L).

Similarly, we found that knockdown of PKM2 significantly reduced *SERPINE1* transcription compared with the negative control (NC) group in MDA-MB-231 cells (Fig. 4M and N). The proliferative, migratory, and invasive capabilities of PKM2-depleted MDA-MB-231 cells could be partially rescued by ectopic expression of PAI-1 (Fig. 4O, P, Q, and R). Consistent experimental results were obtained regarding the roles of NONO and PKM2 in human BT-549 TNBC cells (Figure S2A-L). However, we cannot rule out the possibility that other factors downstream of the NONO/PKM2 pathway may also contribute to the proliferative, migratory, and invasive capabilities of TNBC cells.

To further investigate the clinical relevance of PAI-1 expression in patients with TNBC, we examined its expression using IHC on tissue microarrays containing 60 pairs of TNBC samples and their matched adjacent normal tissues. We demonstrated that PAI-1 was notably upregulated in TNBC tissues compared to adjacent normal tissues (Figure S2M and N). Importantly, the expression levels of NONO and PAI-1, as well as those of PKM2 and PAI-1, were significantly positively correlated in human TNBC samples according to Pearson correlation analysis (Fig. 4S), further confirming that *SERPINE1* is the downstream target gene of NONO/PKM2 in TNBC cells. Collectively, these data suggest that NONO/PKM2 promotes TNBC progression at least in part through the activation of *SERPINE1* transcription.

### NONO-dependent PKM2 and its nuclear protein kinase activity are critical for *SERPINE1* transcription and TNBC progression

**Fig. 5:**
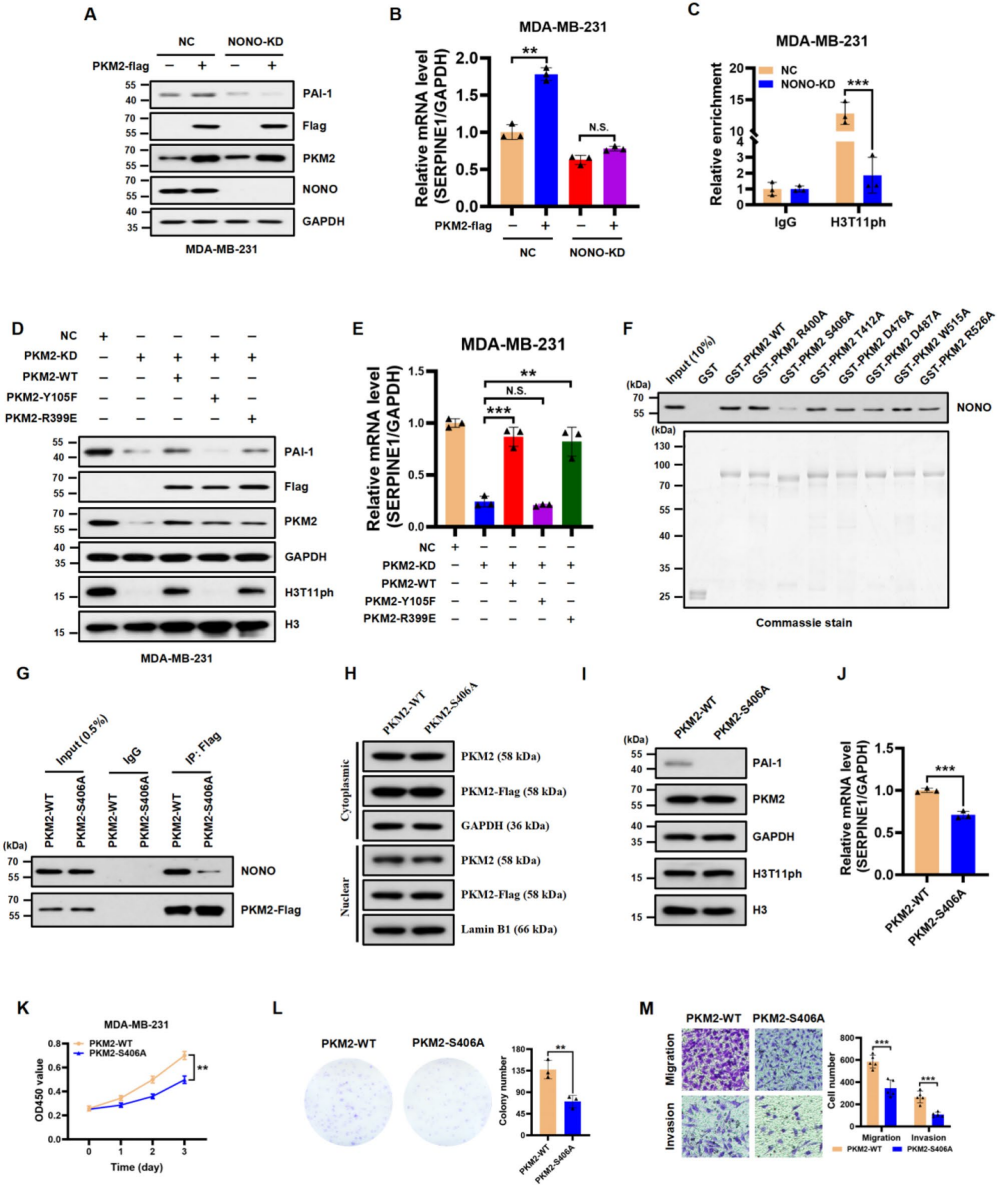
NONO-dependent PKM2 and its nuclear protein kinase activity are critical for *SERPINE1* transcription and TNBC progression. **A** and **B.** The protein and mRNA expression of PAI-1 was assessed by western blot (**A**) and RT‒qPCR (**B**) analyses in NC and NONO-KD MDA-MB-231 cells with or without PKM2 overexpression. GAPDH served as a loading control. The data are presented as the mean ± SD (*n* = 3). **P < 0.01, N.S. (not significant) compared to the corresponding control. **C.** ChIP‒qPCR was used to assess the enrichment of H3T11ph on *SERPINE1* at the TSS in NC and NONO-KD MDA-MB-231 cells. IgG served as a negative control. The data are presented as the mean ± SD (*n* = 3). ***P < 0.001 compared to the NC group. **D.** Western blot analysis of the indicated protein levels in NC cells, PKM2-KD cells, PKM2-KD + PKM2-WT cells, PKM2-KD + PKM2-Y105F cells and PKM2-KD + PKM2-R399E cells. GAPDH served as a loading control. **E.** RT‒qPCR analysis of the mRNA levels of *SERPINE1* (normalized to the level of *GAPDH*) in NC cells, PKM2-KD cells, PKM2-KD + PKM2-WT cells, PKM2-KD + PKM2-Y105F cells and PKM2-KD + PKM2-R399E cells. The data are presented as the mean ± SD (*n* = 3). **P < 0.01, ***P < 0.001, N.S. (not significant) compared to the corresponding control. **F.** A GST pull-down assay was utilized to examine the binding of prokaryotic GST, wild-type (WT) GST-PKM2 and GST-PKM2 point mutants with purified His-NONO fusion proteins (top). GST, GST-PKM2 WT, and diverse GST-PKM2 point mutants were visualized by Commassie staining (bottom). **G.** Endogenous NONO was coimmunoprecipitated in MDA-MB-231 cells overexpressing Flag-tagged PKM2-WT and PKM2-S406A. IgG served as the negative control. **H.** The distribution of endogenous PKM2 and exogenous PKM2-Flag proteins in the nucleus and cytoplasm in MDA-MB-231 cells overexpressing Flag-tagged PKM2-WT and PKM2-S406A was examined by western blot. GAPDH and Lamin B1 served as loading controls. **I.** Western blot analysis of the indicated proteins in PKM2-WT- and PKM2-S406A-overexpressing MDA-MB-231 cells. GAPDH and histone H3 served as loading controls. **J.** RT‒qPCR analysis of the mRNA levels of *SERPINE1* (normalized to the level of *GAPDH*) in MDA-MB-231 cells overexpressing PKM2-WT or the PKM2-S406A mutant. The data are presented as the mean ± SD (*n* = 3). ***P < 0.001. **K.** Cell proliferation was examined by CCK-8 assays in MDA-MB-231 cells overexpressing PKM2-WT or the PKM2-S406A mutant. **L.** Colony formation assay of MDA-MB-231 cells overexpressing PKM2-WT and the PKM2-S406A mutant. The number of colonies is shown in the bar graph (right panels). The data are presented as the mean ± SD (*n* = 3). **P < 0.01 compared to the PKM2-WT group. **M.** Representative images of the migration (top panels) and invasion (bottom panels) of MDA-MB-231 cells overexpressing PKM2-WT or the PKM2-S406A mutant. The data are presented as the mean ± SD (*n* = 5). ***P < 0.001 compared to the corresponding control

**Fig. 6:**
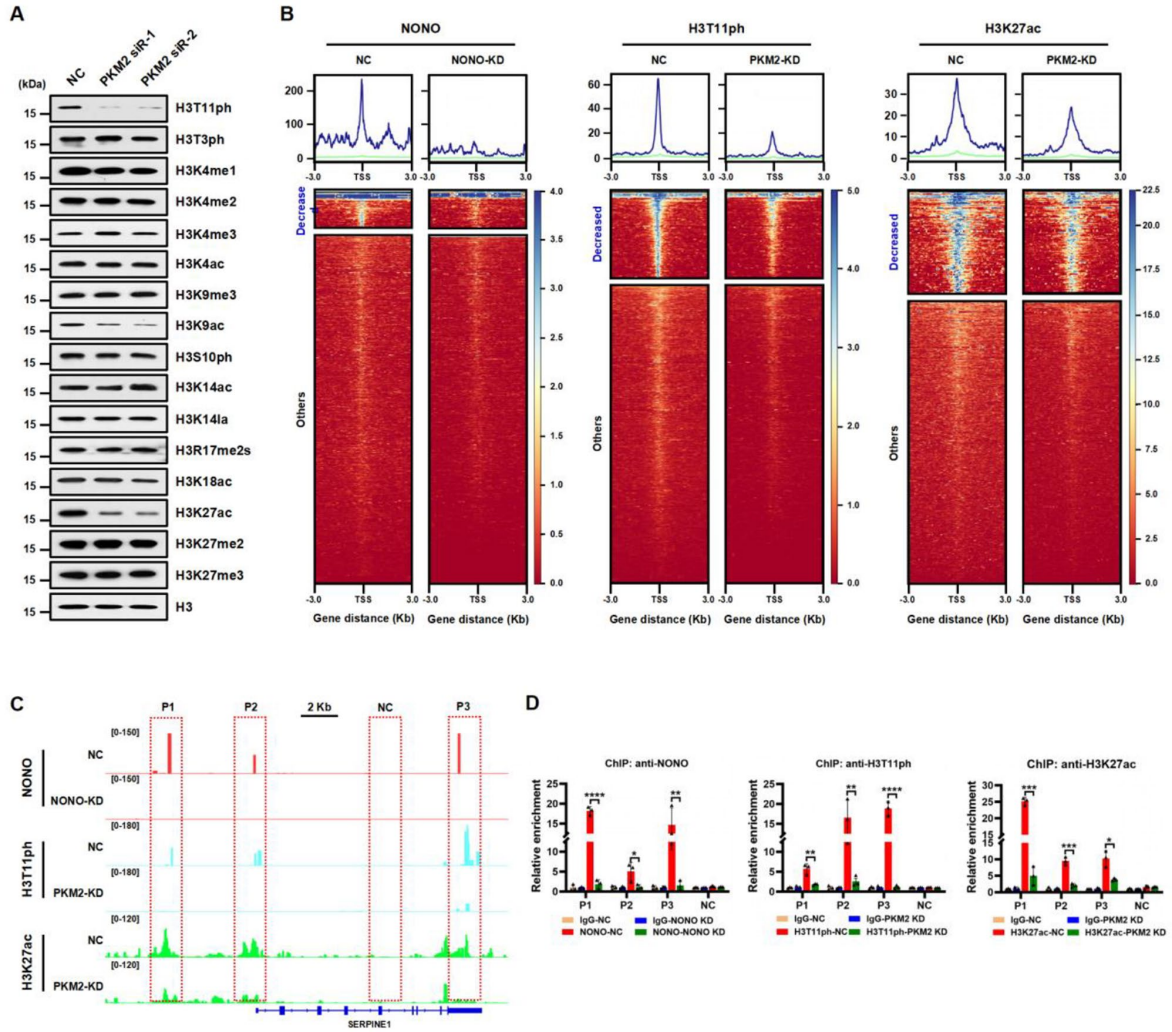
NONO and PKM2-mediated H3T11ph coordinate with H3K27ac on *SERPINE1* gene loci. **(A)** Western blot analysis of the indicated histone H3 modifications in NC and PKM2-silenced MDA-MB-231 cells. Histone H3 served as a loading control. **(B)** Heatmaps of CUT&Tag data showing that NONO, H3T11ph, and H3K27ac levels at the transcription start site (TSS) significantly decreased (Decreased) or unchanged (Others) in the NC, NONO-KD, or PKM2-KD MDA-MB-231 cells, respectively. **(C)** Integrative Genomics Viewer (IGV) tracks representing the signals of NONO, H3T11ph, and H3K27ac at *SERPINE1* gene loci from CUT&Tag data in MDA-MB-231 cells. **(D)** ChIP‒qPCR analysis of the enrichment of NONO, H3T11ph, and H3K27ac at *SERPINE1* gene loci at the indicated positions as in (**C)** in MDA-MB-231 cells. IgG served as a negative control. The data are presented as the mean ± SD (*n* = 3). *P < 0.05, **P < 0.01, ***P < 0.001, and ****P < 0.0001 compared to the corresponding control

Given that NONO and PKM2 positively regulate *SERPINE1* transcription in TNBC cells, and that they directly interact in the nucleus, we investigated how they regulate *SERPINE1* expression. As expected, enforced the overexpression of PKM2 enhanced *SERPINE1* expression. However, this increase in expression was abolished by NONO knockdown (Fig. 5A and B). Next, we examined whether PKM2-mediated H3T11ph modifications at the *SERPINE1* promoter were enriched in a NONO-dependent manner. ChIP‒qPCR revealed a significant reduction in H3T11ph enrichment at the *SERPINE1* promoter in the NONO knockdown group compared to that in the negative control (NC) group (Fig. 5C). Previous studies have demonstrated that PKM2 exhibits pyruvate kinase or protein kinase activity, which depends on its cytosolic tetramer or nuclear dimer state [19, 41, 42]. To further explore whether the protein kinase of PKM2 is crucial for *SERPINE1* transcriptional activation, we utilized two well-established PKM2 mutants, PKM2-Y105F (predominant tetramer form harboring pyruvate kinase activity) [42] and PKM2-R399E (predominant dimer form harboring protein kinase activity) [19] to examine their effects on *SERPINE1* expression. We re-expressed the wild-type PKM2 (PKM2-WT), the Y105F mutant (PKM2-Y105F), or the R399E mutant (PKM2-R399E) in MDA-MB-231 cells with PKM2 knockdown, using synonymous codon mutations to make these constructs resistant to siRNA-mediated degradation. We found that the PKM2-Y105F mutant did not activate *SERPINE1* expression, whereas the PKM2-R399E mutant had an activating effect on *SERPINE1* expression similar to that of PKM2-WT (Fig. 6D and E). These results suggest that the protein kinase activity of PKM2 is key to activating *SERPINE1* expression in the nucleus.

To identify the potential key residues of PKM2 responsible for NONO interactions, we used the ZDOCK server (https://zdock.umassmed.edu/) for molecular docking analyses. Molecular docking confirmed good and stable binding of NONO to PKM2 (Figure S3A) and revealed seven positions of PKM2 that may play important roles in the interaction between PKM2 and NONO (Figure S3B). GST pull-down assays demonstrated that the PKM2-S406A mutant (Ser to Ala at aa 406) significantly reduced this interaction, whereas the interactions of PKM2-R400A, T412A, D476A, D487A, W515A, and R526A mutants with NONO were similar to those of wild-type PKM2 (PKM2-WT, Fig. 5F). To explore the effect of the S406A mutation of PKM2 on TNBC progression, we overexpressed PKM2-WT or the PKM2-S406A mutant in MDA-MB-231 cells. Consistently, the PKM2-S406A mutant immunoprecipitated significantly less NONO in the MDA-MB-231 cells (Fig. 5G). Notably, the PKM2-S406A mutant exhibited the same subcellular distribution as that of PKM2-WT (Fig. 5H). However, compared with PKM2-WT, PKM2-S406A overexpression in MDA-MB-231 cells failed to activate *SERPINE1* expression (Fig. 5I and J). In addition, we found that compared to PKM2-WT, PKM2-S406A overexpression in MDA-MB-231 cells significantly inhibited cell growth, migration, and invasion (Fig. 5K, L, and M). Taken together, these results demonstrated that NONO-dependent PKM2 and its nuclear protein kinase activity are crucial for *SERPINE1* expression and TNBC cell invasion.

### PKM2-mediated H3T11ph cooperates with TIP60-mediated H3K27ac to promote *SERPINE1* expression

A previous study has revealed that PKM2 mediates H3T11ph, which is involved in transcriptional regulation [21]. To explore the effect of PKM2 on other histone modifications, we examined global changes in other key histone H3 modifications upon PKM2 knockdown in MDA-MB-231 cells. As expected, the levels of H3T11ph were markedly decreased upon PKM2 knockdown (Fig. 6A). Interestingly, we found that the levels of H3K27ac, a promoter and enhancer marker [43–45], were markedly lower in PKM2-knockdown cells than in NC cells (Fig. 6A). Additionally, we observed that the levels of the histone markers H3K4me1 and H3K9ac, which have been previously described [21], decreased upon PKM2 knockdown (Fig. 6A). There were no obvious changes in the levels of H3T3ph, H3K4me2/3, H3K4ac, H3K9me3, H3S10ph, H3K14ac, H3K14la, H3R17me2s, H3K18ac, or H3K27me2/3 modifications between PKM2 knockdown cells and NC cells (Fig. 6A). These data suggested that PKM2-mediated H3T11ph cooperates with H3K27ac to regulate gene transcription in TNBC cells.

To understand how NONO, H3T11ph, and H3K27ac collaborate to regulate gene transcription, we performed cleavage under targets and tagmentation (CUT&Tag) experiments in MDA-MB-231 cells to determine the enrichment profiles of NONO, H3T11ph, and H3K27ac in the whole genome. Intriguingly, our analysis revealed that the signals for NONO, H3T11ph, and H3K27ac were significantly enriched at transcription start sites (TSSs) (Fig. 6B, GSE266179). This enrichment suggests that these factors may play important roles in transcriptional regulation. The GSEA enrichment analysis was employed to evaluate the pathways regulated by NONO, H3T11ph, and H3K27ac respectively (Figure S4A). In addition, bioinformatic analysis demonstrated that H3T11ph and H3K27ac signals were markedly reduced in PKM2-KD cells compared to their respective NC cells (Fig. 6B). In fact, we found that NONO, H3T11ph, and H3K27ac were co-enriched in the promoter, TSS, and 3’-end regions of *SERPINE1* (Fig. 6C). These results were further confirmed by ChIP‒qPCR in MDA-MB-231 cells (Fig. 6D). Notably, NONO enrichment largely overlapped with H3T11ph peaks genome-wide in MDA-MB-231 cells (Figure S4B, GSE266179). Moreover, through bioinformatics analysis, we found that the genome-wide occupancy of NONO and H3T11ph, H3T11ph, and H3K27ac was highly correlated, although there was no significant correlation between NONO and H3K27ac (Figure S4C). These results further suggest that PKM2-mediated H3T11ph could be enriched in promoters through NONO recruitment of PKM2, where H3T11ph cooperates with H3K27ac to activate *SERPINE1* transcription.

Next, we explored the possible link between H3T11ph and H3K27ac. We searched the RNA-seq data obtained upon PKM2 knockdown (GSE266177) and examined changes in the expression levels of histone acetyltransferases *KAT5* (encoding TIP60), *KAT3B* (encoding PCAF), *CREBBP* (encoding CBP), and *EP300* (encoding P300), which are associated with histone acetylation. We then performed western blot analyses and found that PKM2 knockdown significantly reduced the expression of TIP60 but upregulated P300 expression in MDA-MB-231 cells, whereas the expression of PCAF and CBP remained unchanged compared with that in NC controls (Figure S5A). Therefore, we selected KAT5/TIP60 for further study. RT-qPCR assays confirmed that *KAT5* mRNA levels significantly decreased following PKM2 knockdown (Figure S5B). Similar results were obtained for the BT-549 cells (Figure S6A and B). In addition, our CUT&Tag data demonstrated that H3T11ph modifications were enriched in the *KAT5* promoter and that H3T11ph enrichment was significantly reduced in the promoter upon PKM2 knockdown (Figure S5C). Consistent results were obtained by ChIP‒qPCR in MDA-MB-231 cells (Figure S5D). These data indicated that PKM2 directly activates *KAT5* transcription.

To determine whether TIP60 regulates *SERPINE1* expression, we knocked down *KAT5* expression in MDA-MB-231 cells. *KAT5* knockdown significantly reduced *SERPINE1* expression in MDA-MB-231 cells compared to that in NC control cells (Figure S5E and F). As expected, the levels of H3K27ac were markedly reduced following *KAT5* knockdown (Figure S5F). Importantly, the levels of PAI-1 and H3K27ac in PKM2-depleted MDA-MB-231 cells were partially restored by ectopic TIP60 expression (Figure S5G). These results were also observed in BT-549 cells (Figure S6C, D and E). In addition, we analyzed the expression profiles extracted from GEO datasets (GSE76275) and found that the expression of *PKM2* and *KAT5* was positively correlated in human TNBC samples (Figure S6F). Taken together, these results suggest that PKM2 can also transcriptionally regulate *KAT5* expression and that TIP60-mediated H3K27ac cooperates with PKM2-mediated H3T11ph to activate *SERPINE1* expression.

### NONO or PKM2 deficiency reduces PAI-1 expression and inhibits the malignant progression of spontaneous mammary tumors in mice

**Fig. 7:**
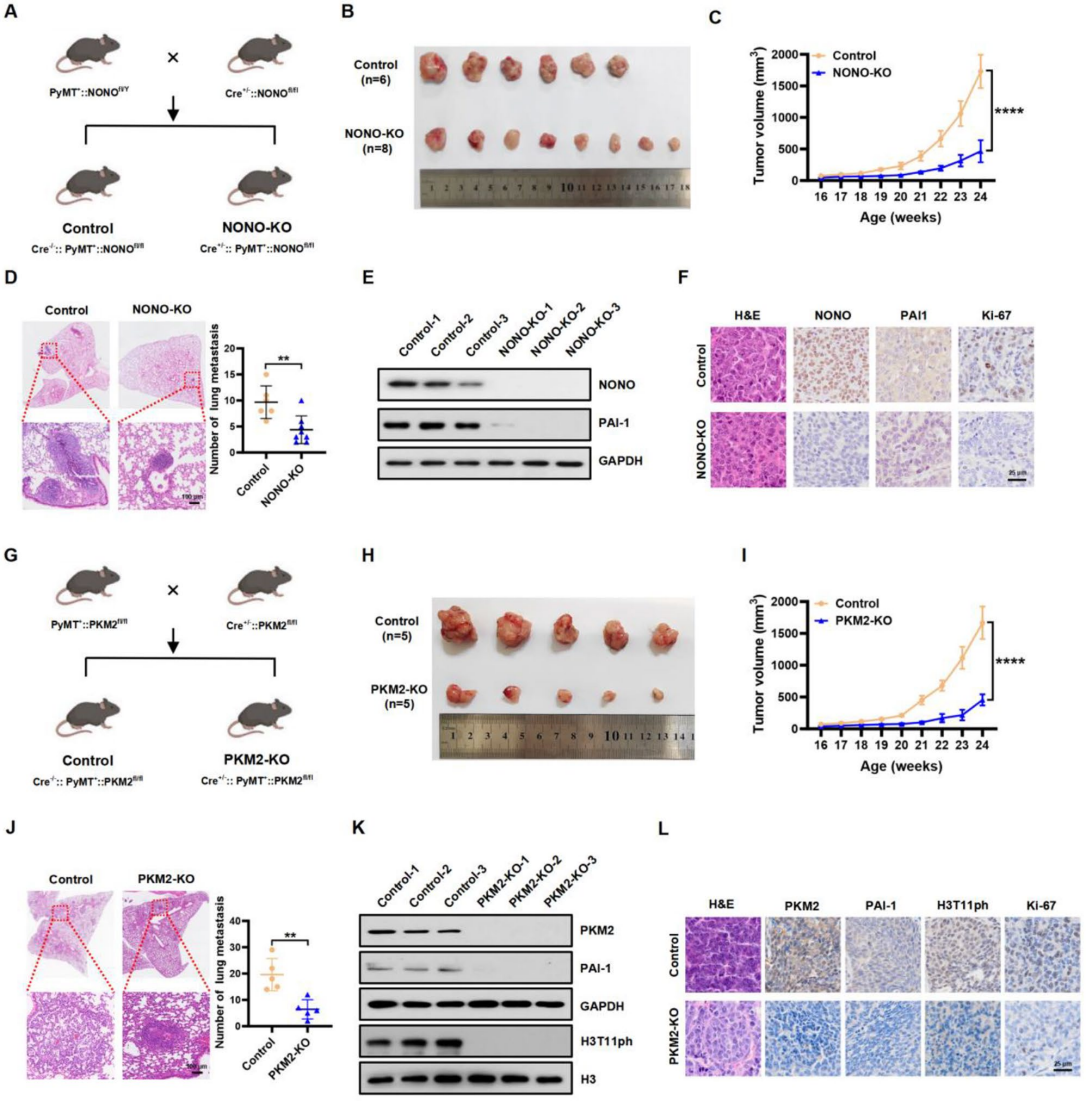
NONO or PKM2 deficiency reduces PAI-1 expression and inhibits the malignant progression of spontaneous mammary tumors in mice. **A.** Breeding strategy for mammary-specific NONO-knockout (KO) transgenic mice. The genotype of the NONO-KO female mice was Cre^+/−^::PyMT^+^::NONO^fl/fl^, and Cre^−/−^::PyMT^+^::NONO^fl/fl^ female mice were used as control mice. **B-D.** Representative images of excised tumors (**B**), tumor growth curves (**C**), representative H&E staining images of lungs (left panels) and the number of lung metastases (right panels) (**D**) from control (*n* = 6) and NONO-KO (*n* = 8) mice. Scale bar, 100 μm. All data are presented as the mean ± SD. **P < 0.01 compared to the control group. **E.** Western blot analysis of the indicated proteins in tumors extracted from control and NONO-KO mice. GAPDH served as a loading control. **F.** Representative H&E staining and IHC staining of NONO, PAI-1, and Ki-67 in mammary tumor tissues from MMTV-PyMT mice (C57BL/6 background) in the control and NONO-KO groups. Scale bar, 25 μm. **G.** Breeding strategy for mammary-specific PKM2-knockout (KO) transgenic mice. The genotype of the PKM2-KO female mice was Cre^+/−^::PyMT^+^::PKM2^fl/fl^, and Cre^−/−^::PyMT^+^::PKM2^fl/fl^ female mice were used as control mice. **H-J.** Representative images of excised tumors (**H**), tumor growth curves (**I**), representative H&E staining images of lungs (left panels) and the number of lung metastases (right panels) (**J**) from control (*n* = 5) and PKM2-KO (*n* = 5) mice. Scale bar, 100 μm. All data are presented as the mean ± SD. **P < 0.01 compared to the control group. **K.** Western blot analysis of the indicated proteins in tumors extracted from control and PKM2-KO mice. GAPDH and histone H3 served as loading controls. **L.** Representative H&E staining and IHC staining of PKM2, PAI-1, H3T11ph and Ki-67 in mammary tumor tissues from MMTV-PyMT mice in the control and PKM2-KO groups. Scale bar, 25 μm

The nuclear protein NONO is highly conserved in humans and mice, and shares 98% amino acid sequence similarity [7, 8]. To further assess the role of NONO in regulating mammary tumor progression in vivo, we constructed a spontaneous mammary tumor mouse line in which NONO was specifically knocked out in the breast tissue. To obtain NONO knockout (MMTV-Cre^+/−^::MMTV-PyMT^+^::NONO^fl/fl^) female mice, MMTV-Cre^+/−^::NONO^fl/fl^ female mice were crossed with the male MMTV-PyMT^+^::NONO^fl/Y^ mice. Female littermates (MMTV-Cre^−/−^::MMTV-PyMT^+^::NONO^fl/fl^) were used as controls (Fig. 7A). Consistent with our previous results, conditional loss of NONO in mammary tissue significantly inhibited tumor growth (Fig. 7B and C; Figure S7A). In addition, knockout of NONO markedly reduced the lung metastatic capacity and suppressed the formation of larger metastatic nodules in spontaneous mammary tumor model mice (Fig. 7D). The intratumoral levels of NONO, PAI-1, and Ki-67 were analyzed using western blotting and immunohistochemical (IHC) staining. Conditional mammary NONO depletion significantly reduced PAI-1 and Ki-67 expression (Fig. 7E and F; Figure S7B and C).

We also generated a mouse model of spontaneous breast cancer with mammary-specific PKM2 knockout. To obtain PKM2 knockout (MMTV-Cre^+/−^::MMTV-PyMT^+^::PKM2^fl/fl^) female mice, MMTV-Cre^+/−^::PKM2^fl/fl^ female mice were crossed with MMTV-PyMT^+^::PKM2^fl/fl^ male mice. Female littermates (MMTV-Cre^−/−^::MMTV-PyMT^+^::PKM2^fl/fl^) were used as controls (Fig. 7G). Similarly, tumor growth and lung metastatic capacity were significantly inhibited by conditional PKM2 knockout in breast tissue (Fig. 7H, I, and J; Figure S7D). The intratumoral levels of PKM2, PAI-1, and Ki-67 were analyzed using western blotting and immunohistochemical (IHC) staining. Conditional mammary PKM2 depletion significantly reduced PAI-1 and Ki-67 expression (Fig. 7K and L; Figure S7E and F). Taken together, these in vivo data reinforce the notion that NONO and PKM2 are critical for the malignant progression of breast tumors via *SERPINE1* transcriptional activation.

## Discussion

**Fig. 8:**
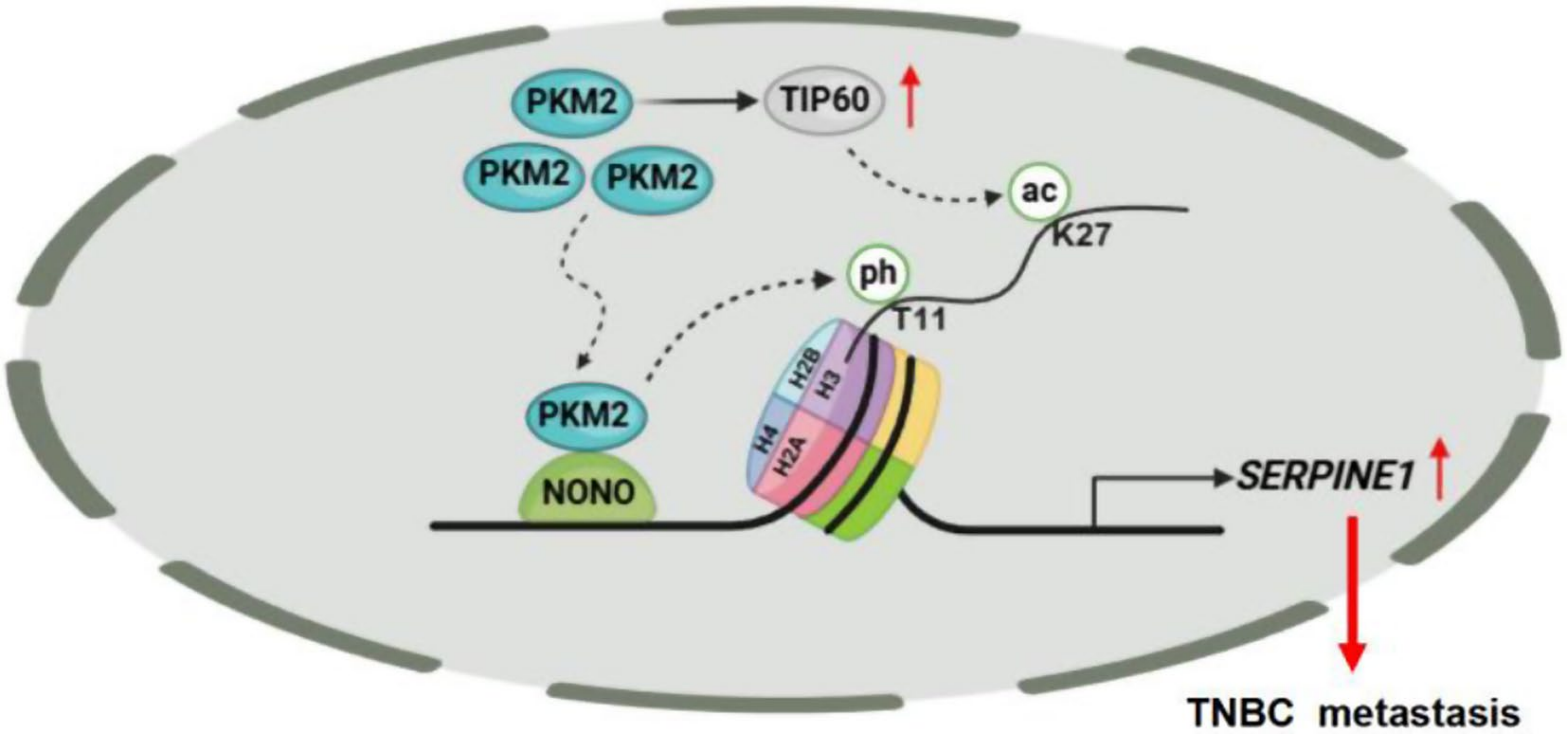
The schematic diagram illustrates the mechanism by which the interaction between NONO and nuclear PKM2 promotes TNBC metastasis. NONO interacts with nuclear PKM2, and PKM2-mediated H3T11ph cooperates with TIP60-mediated H3K27ac to activate SERPINE1 transcription, thereby promoting cell metastasis in TNBC

The prognosis of patients with TNBC remains poor because of the lack of specific biomarkers for effective interventions [2, 46]. Therefore, elucidating novel molecular mechanisms and developing effective therapeutic targets for TNBC is highly important. In this study, we found that NONO specifically interacts with nuclear PKM2 to transcriptionally activate *SERPINE1* expression and promote TNBC cell metastasis. In addition, we found that PKM2-mediated H3T11ph cooperates with TIP60-mediated H3K27ac to regulate gene transcription in an NONO-dependent manner (Fig. 8). These results revealed a novel mechanism by which PKM2, a protein kinase, directly regulates gene transcription and promotes TNBC metastasis.

As a multifunctional nuclear protein, NONO has diverse functions in tumorigenesis, through which it may interact with distinct protein partners [12]. Previous studies have shown that NONO interacts with the Hippo pathway effector TAZ to undergo liquid–liquid phase separation, which promotes TAZ-mediated oncogenic transcription [47]. In addition, a recent study demonstrated that NONO is important for transactivation of EGFR by stabilizing nuclear EGFR expression and recruiting other transcriptional coactivators [14]. However, how the NONO-driven transcriptional program facilitates malignant development of cancer remains unclear. In this study, we found that PKM2 directly interacted with NONO and executed its protein kinase function to facilitate TNBC metastasis. Our data showed that NONO enrichment was strongly correlated with nuclear PKM2-mediated H3T11ph marks along genomic loci, including *SERPINE1*. Interestingly, we observed that approximately 45% of the NONO-targeting genes were also regulated by PKM2 in TNBC cells. These findings suggested that nuclear PKM2 is important for NONO-mediated gene expression in TNBC cells.

In addition to its glycolytic function, PKM2 has non-metabolic functions that are critical for gene expression. Within the nucleus, PKM2 is a transcriptional coactivator that confers malignant potential to cancer cells through its association with various transcription factors including Oct-4, HIF-1α, β-catenin, and STAT3 [19, 20, 48, 49]. However, the regulatory processes associated with non-metabolic PKM2 involve non-histone phosphorylation (H3T11ph)-mediated transcription of downstream target genes. Intriguingly, upon EGF receptor activation, PKM2 binds histone H3 and phosphorylates histone H3 at T11, resulting in the acetylation of histone H3 at K9 to activate CCND1 and MYC expression in glioblastoma cells [21]. In addition, a recent study reported that PKM2 interacts with the histone methyltransferase EZH2 to regulate the metabolic switch from glycolysis to fatty acid βoxidation in TNBC [30]. However, there was no indication of how PKM2/EZH2 was recruited to the target genes in this study [30]. In contrast, we did not observe any change in H3K27me3 levels upon PKM2 knockdown compared with the NC control in MDA-MB-231 cells in our study. This discrepancy could be due to different cell sources and manipulations. Interestingly, in the current study, we found that PKM2 phosphorylated histone H3 at T11 in a NONO-dependent manner, and this phosphorylation coincided with the acetylation of histone H3 at K27 to activate *SERPINE1* expression in TNBC. These results revealed more precise genome-wide enrichment of PKM2-mediated H3T11ph and demonstrated a novel crosslink between histone marks. H3K27ac is an active enhancer mark [43–45]. Thus, our data suggests that PKM2-mediated H3T11ph may also be associated with active enhancers. NONO can bind to enhancer regions in human glioblastoma cells and mouse retina [47, 50]. Therefore, the mechanism by which PKM2-mediated H3T11ph modification is linked to the NONO-mediated regulation of enhancer activity warrants further investigation. Nevertheless, the specific role of PKM2 makes it an attractive therapeutic target in cancer treatment. However, the complexity of PKM2 regulation and signaling has led researchers to face the following dilemma: whether to use PKM2 activators or inhibitors for cancer treatment [17]. The current study provides an alternative intervention strategy for treating TNBC by inhibiting or interfering with the interaction between NONO and PKM2.

PAI-1 is a major member of the serine protease inhibitor (serpin) superfamily and functions as an inhibitor of tissue-type plasminogen activator (tPA) and urokinase-type plasminogen activator (uPA) [51]. tPA/uPA is mainly involved in the conversion of plasminogen to active plasmin, leading to fibrin clot hydrolysis [51]. Therefore, PAI-1 plays an important role in various vascular disorders such as thrombosis, atherosclerosis, and myocardial infarction [52]. Interestingly, accumulating evidence has demonstrated that PAI-1 plays a pro-tumorigenic role in cancer, although it was originally hypothesized to have antitumor effects [53]. Owing to the strong correlation between PAI-1 levels and prognosis in patients with breast cancer, PAI-1 has been recommended as a biomarker for therapeutic decisions in patients with clinically node-negative breast cancer [54]. Several studies have shown that PAI-1 is critical for TNBC cell growth and metastasis [32, 55, 56]. In the present study, we demonstrated that NONO can recruit nuclear PKM2, which triggers the phosphorylation of histone H3 at T11 to directly activate *SERPINE1* expression and mediate cell proliferation, migration, and invasion in TNBC cells. More importantly, NONO and PKM2 expression strongly correlated with the expression of PAI-1 in TNBC tissues. These findings identified a novel transcriptional regulatory pathway for *SERPINE1* expression and support the function of the NONO-PKM2 interaction in the malignant progression of TNBC.

## Conclusions

Taken together, our findings demonstrate that NONO interacts with nuclear PKM2 and directs histone H3 phosphorylation to promote tumor metastasis, highlighting the potential of disrupting the association between NONO and PKM2 for the targeted therapy of malignant TNBC.

## Electronic supplementary material

Below is the link to the electronic supplementary material.

Supplementary Material 1

## Data Availability

Raw mass spectrometry proteomics data were deposited in the ProteomeXchange consortium under the accession number PXD051842. The RNA-Seq and CUT&Tag data generated for this study were deposited in the Gene Expression Omnibus (GSE266177, GSE266179).

## Abbreviations

NONO: Non-POU domain-containing octamer-binding protein
PKM2: Pyruvate kinase isozyme type M2
H3T11ph: Phosphorylation of histone H3 at threonine 11
H3K27ac: Acetylation of histone H3 at lysine 27
SERPINE1: Serine protease inhibitor family E member 1
PAI-1: Plasminogen Activator Inhibitor 1
TNBC: Triple-negative breast cancer
ChIP: Chromatin immunoprecipitation
CUT&Tag: Cleavage under targets and tagmentation
TSS: transcription start sites
IHC: immunohistochemical staining
DEG: Differentially expressed genes
TIP60: HIV-tat interacting protein 60kD
