# Supplementary material for: NONO interacts with nuclear PKM2 and directs histone H3 phosphorylation to promote triple-negative breast cancer metastasis
Source: J Exp Clin Cancer Res. 2025 Mar 10;44:90. doi: 10.1186/s13046-025-03343-5 (PMC11892261; doi:10.1186/s13046-025-03343-5)

Supplementary data to

**NONO interacts with nuclear PKM2 and directs histone H3 phosphorylation to promote triple-negative breast cancer metastasis**

**Figure S1. Expression levels of NONO or PKM2 in human breast cancer tissues from the Gene Expression Omnibus repository GSE76275.**

**(A)** Quantitative analysis of NONO expression levels in non-TNBC (n = 67) and TNBC (n = 198) tissue samples. The data are presented as the mean ± SD. ****P* < 0.001. **(B)** Quantitative analysis of PKM2 expression levels in non-TNBC (n = 67) and TNBC (n = 198) tissue samples. The data are presented as the mean ± SD. ****P* < 0.001.


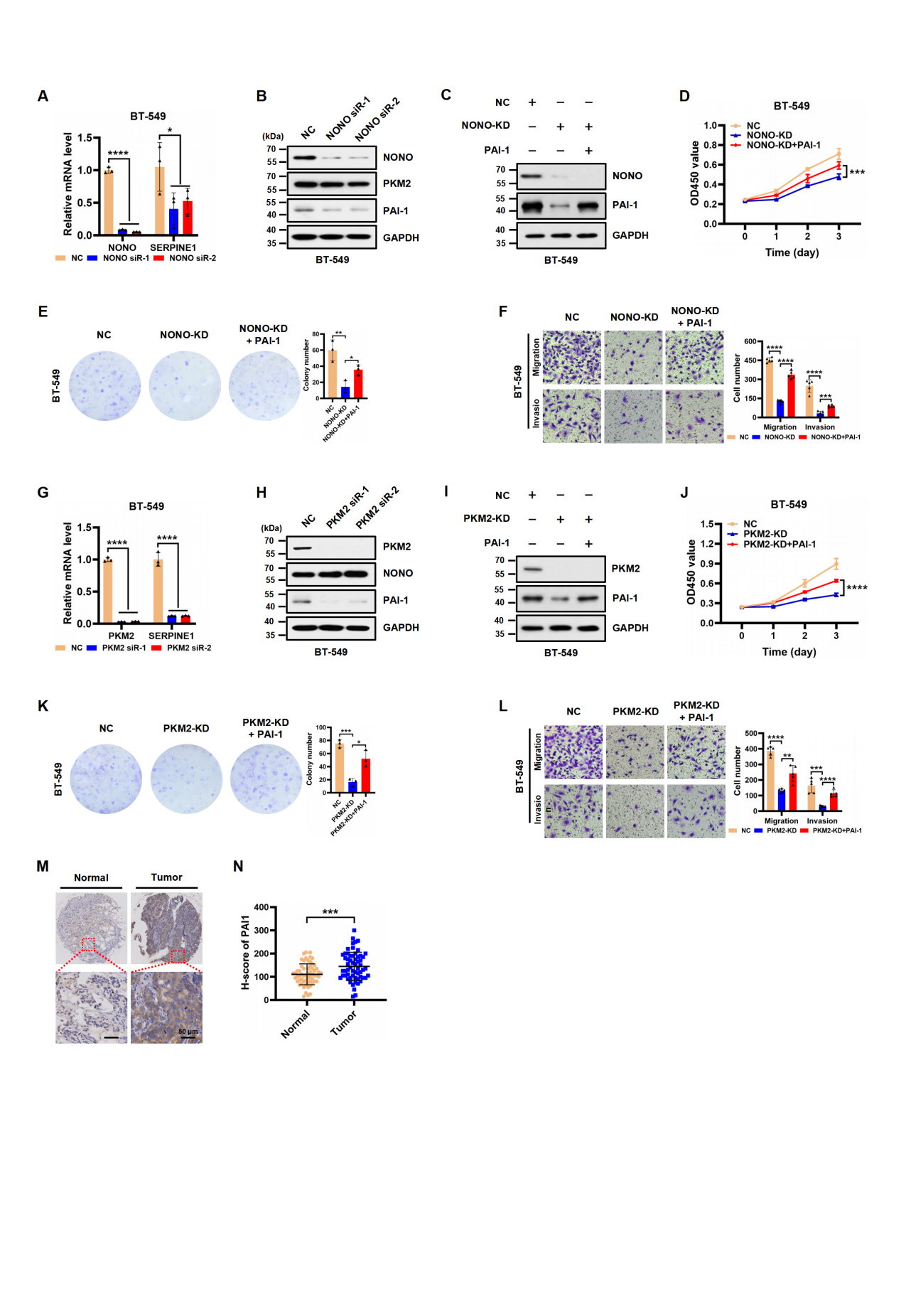


**Figure S2. NONO and PKM2 promote cell proliferation and metastasis by upregulating** ***SERPINE1* transcription in BT-549 cells.**

**(A)** The relative mRNA level of *SERPINE1* normalized to that of *GAPDH* was examined by RT‒qPCR in NC and NONO-KD BT-549 cells. The data are presented as the mean ± SD (n = 3). **P* < 0.05, *****P* < 0.0001 compared to NC. **(B)** The protein expression of PAI-1 in NC and NONO-KD BT-549 cells was assessed by western blot analyses with the indicated antibodies. **(C)** Western blot analysis of the indicated proteins in BT-549 cells treated with NC, NONO-KD, or NONO-KD + PAI-1. GAPDH served as a loading control. **(D)** Cell proliferation was examined by a CCK-8 assay in BT-549 cells treated with NC, NONO-KD, or NONO-KD + PAI-1. The data are presented as the mean ± SD (n = 5). ****P* < 0.001 compared to the NONO-KD group. **(E)** Colony formation ability of BT-549 cells treated with NC, NONO-KD, or NONO-KD + PAI-1 was determined. The data are presented as the mean ± SD (n = 3). **P* < 0.05, ***P* < 0.01 compared to the corresponding control. **(F)** Representative images of the migration (top panels) and invasion (bottom panels) of BT-549 cells treated with NC, NONO-KD, or NONO-KD + PAI-1. The data are presented as the mean ± SD (n = 5). ****P* < 0.001, *****P* < 0.0001 compared to the corresponding control. **(G)** The relative mRNA level of *SERPINE1* (normalized to that of *GAPDH*) in NC and PKM2-KD BT-549 cells was examined by RT‒qPCR. The data are presented as the mean ± SD (n = 3). *****P* < 0.0001 compared to NC. **(H)** The protein expression of PAI-1 in NC and PKM2-KD BT-549 cells was assessed by western blot analyses with the indicated antibodies. **(I)** Western blot analysis of the indicated proteins in BT-549 cells treated with NC, PKM2-KD, or PKM2-KD + PAI-1. GAPDH served as a loading control. **(J)** Cell proliferation was examined by a CCK-8 assay in BT-549 cells treated with NC, PKM2-KD, or PKM2-KD + PAI-1. The data are presented as the mean ± SD (n = 5). ****P* < 0.001 compared to the PKM2-KD group. **(K)** Colony formation was determined in BT-549 cells treated with NC, PKM2-KD, or PKM2-KD + PAI-1. The data are presented as the mean ± SD (n = 3). **P* < 0.05, ****P* < 0.001 compared with the PKM2-KD group. **(L)** Representative images of the migration (top panels) and invasion (bottom panels) of BT-549 cells treated with NC, PKM2-KD, or PKM2-KD + PAI-1. The data are presented as the mean ± SD (n = 5). ***P* < 0.01, *****P* < 0.0001 compared to the corresponding control. **(M)** Representative images of IHC staining of PAI-1 in matched normal tissues (n = 60) and TNBC tissues (n = 60). Scale bar, 50 μm. **(N)** Quantitative analysis of PAI-1 expression levels in matched normal tissue (Normal) and TNBC tissue (Tumor) samples. The data are presented as the mean ± SD. ****P* < 0.001.


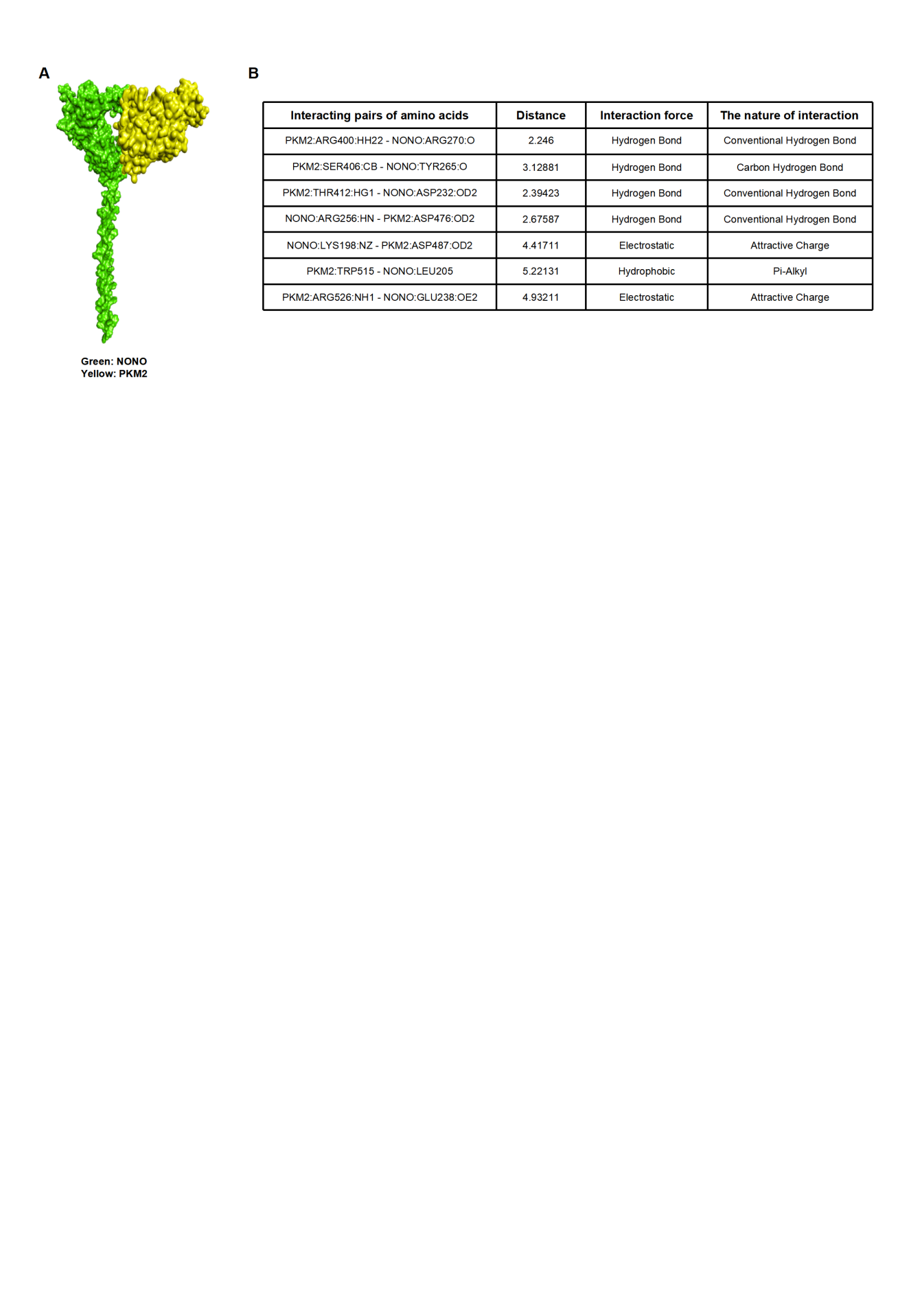


**Figure S3. Prediction of crucial amino acids of PKM2 that interact with NONO.**

**(A)** The molecular docking model for NONO (green) and PKM2 (yellow) was predicted using the ZDOCK server (<https://zdock.umassmed.edu/>). **(B)** Predicted amino acid residues responsible for the interaction between NONO and PKM2.


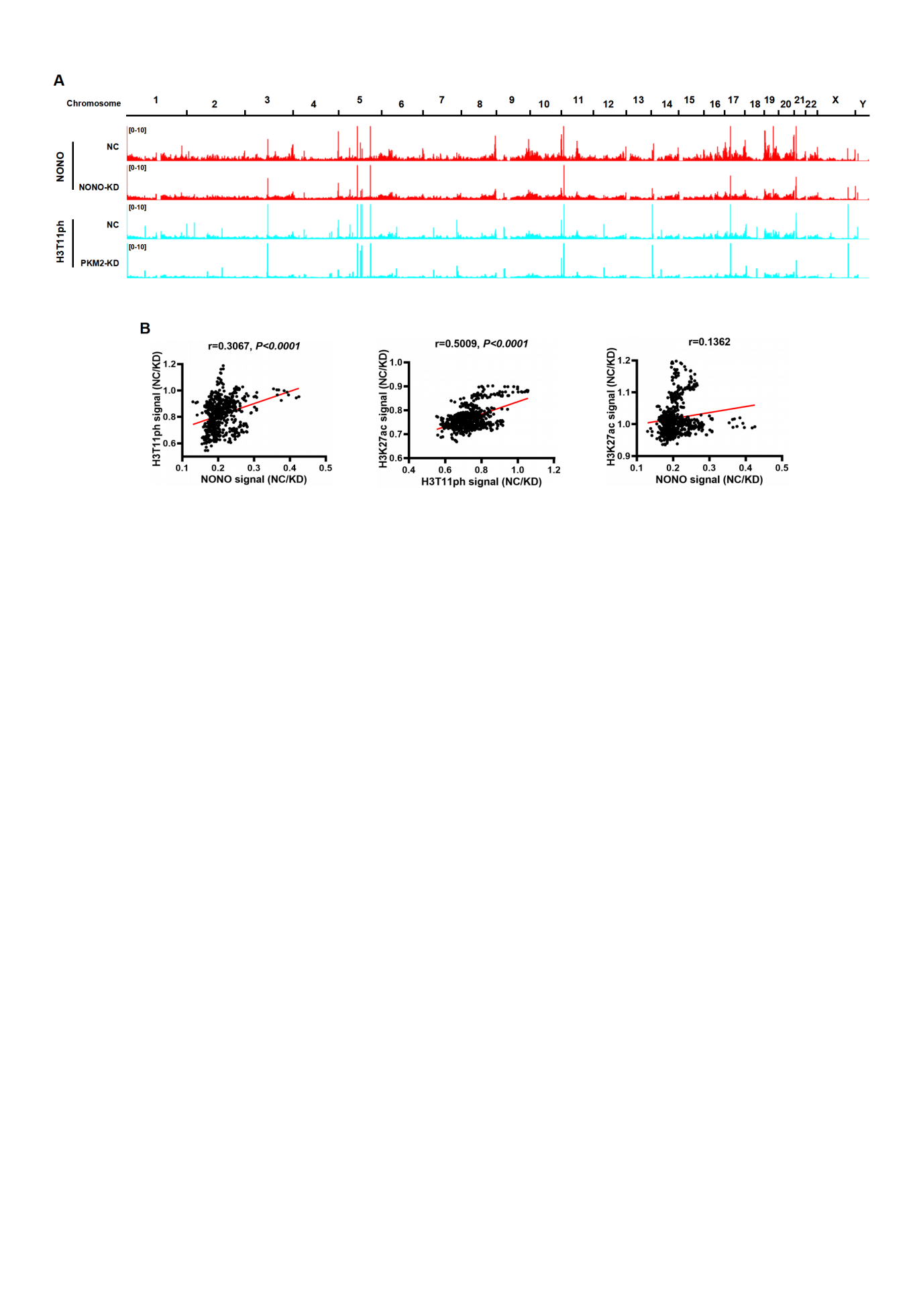


**Figure S4. Genomic tracks of NONO and H3T11ph in MDA-MB-231 cells.**

**(A)** CUT&Tag profiles for NONO enrichment in NONO knockdown and NC control MDA-MB-231 cells, and H3T11ph enrichment in PKM2 knockdown and NC control MDA-MB-231 cells aligned with human chromosomes. **(B)** Correlations between enrichment changes in NONO and H3T11ph, H3T11ph and H3K27ac, or NONO and H3K27ac at promoters in MDA-MB-231 cells. Each dot indicates a single promoter. r, correlation coefficients that were assessed by Pearson product-moment correlation.


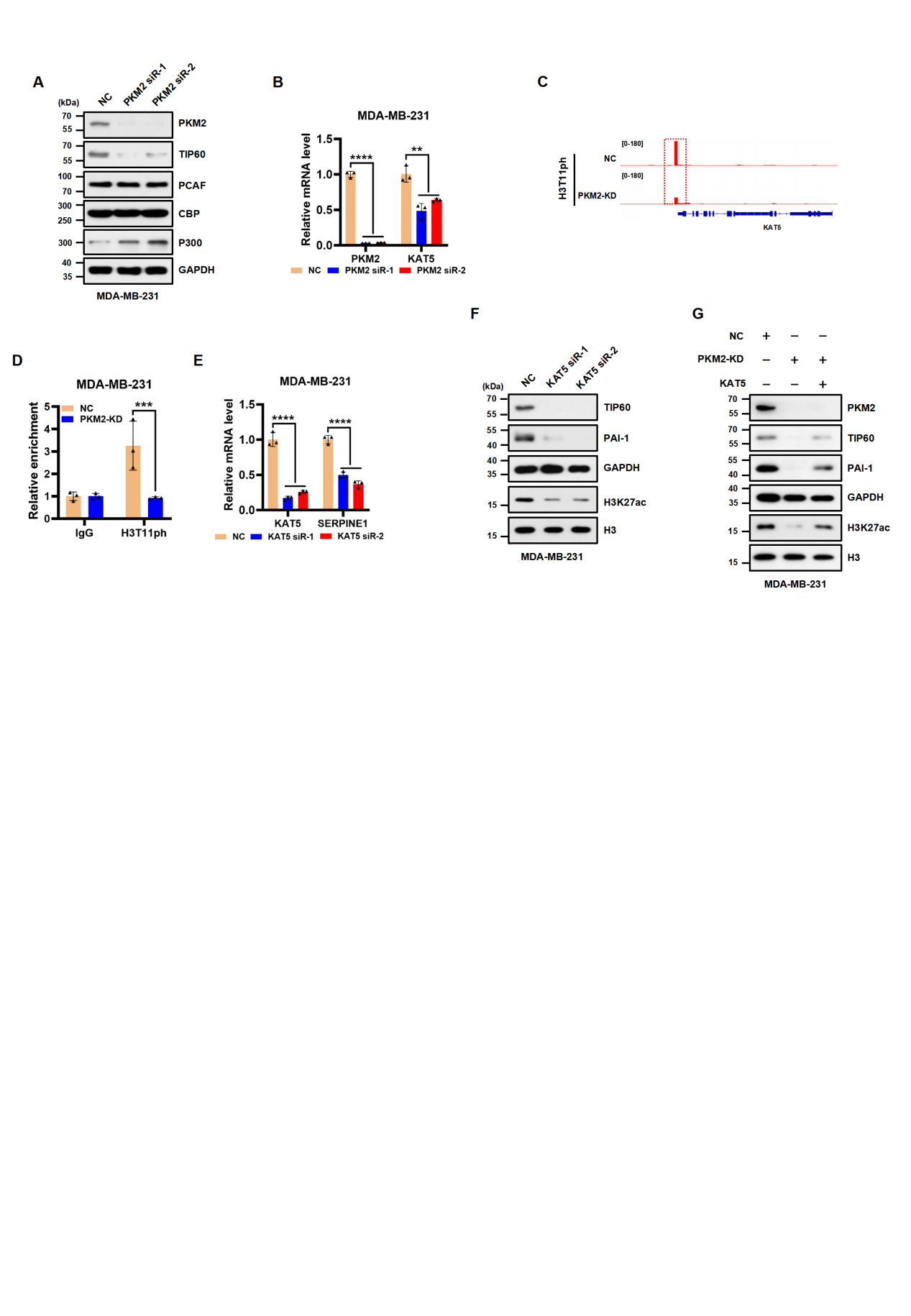


**Figure S5. PKM2 transcriptionally activates *KAT5*, which results in H3K27ac in MDA-MB-231 cells.**

**(A)** Western blot analysis of the indicated proteins in NC and PKM2-knockdown MDA-MB-231 cells. GAPDH served as a loading control. **(B)** The relative mRNA level of *KAT5* normalized to that of *GAPDH* was examined by RT‒qPCR in NC and PKM2-silenced MDA-MB-231 cells. The data are presented as the mean ± SD (n = 3). ***P* < 0.01, *****P* < 0.0001 compared to the NC group. **(C)** IGV tracks showing the enrichment of H3T11ph signals at *KAT5* loci from CUT&Tag data in NC and PKM2-KD MDA-MB-231 cells. **(D)** ChIP‒qPCR was used to assess the enrichment of H3T11ph at *KAT5* loci in NC and PKM2-KD MDA-MB-231 cells. IgG served as a negative control. The data are presented as the mean ± SD (n = 3). ****P* < 0.001 compared to the NC group. **(E)** The relative mRNA level of *SERPINE1* normalized to that of *GAPDH* was examined by RT‒qPCR in NC and TIP60-silenced MDA-MB-231 cells. The data are presented as the mean ± SD (n = 3). *****P* < 0.0001 compared to the NC group. **(F)** Western blot analysis of the indicated proteins in NC and TIP60-KD MDA-MB-231 cells. GAPDH and histone H3 served as loading controls. **(G)** Western blot analysis of the indicated proteins in MDA-MB-231 cells treated with NC, PKM2-KD, or PKM2-KD + TIP60. GAPDH and histone H3 served as loading controls.

**Figure S6. PKM2 transcriptionally activates *KAT5*, which results in H3K27ac in BT-549 cells.**

**(A)** The relative mRNA level of *KAT5* (normalized to that of *GAPDH*) was examined by RT‒qPCR in NC and PKM2-KD BT-549 cells. The data are presented as the mean ± SD (n = 3). ***P* < 0.01, *****P* < 0.0001 compared to the NC group. **(B)** Western blot analysis of the indicated proteins in NC and PKM2-silenced BT-549 cells. GAPDH served as a loading control. **(C)** The relative mRNA level of *SERPINE1* normalized to that of *GAPDH* was examined by RT‒qPCR in NC and KAT5-KD BT-549 cells. The data are presented as the mean ± SD (n = 3). ***P* < 0.01, ****P* < 0.001 compared to the NC group. **(D)** Western blot analysis of the indicated proteins in NC and KAT5-KD BT-549 cells. GAPDH and histone H3 served as loading controls. **(E)** Western blot analysis of the indicated proteins in BT-549 cells treated with NC, PKM2-KD, or PKM2-KD + TIP60. GAPDH and histone H3 served as loading controls. **(F)** Pearson correlation scatter plot of PKM2 and KAT5 in human TNBC tissues (n = 198). The mRNA expression of *PKM2* and *KAT5* was extracted from the GEO dataset GSE76275.

**Figure S7. NONO or PKM2 knockout reduces tumor weight and *SERPINE1* transcription in MMTV-PyMT mice.**

**(A)** Tumor weights of control and NONO-KO mice. All data are the mean ± SD. ***P*< 0.01. **(B)** RT‒qPCR analysis of *SERPINE1* mRNA normalized to *GAPDH* in mammary tumors from control and NONO-KO mice. All data are presented as the mean ± SD. **P*< 0.05. **(C)** Tumor weights of control and PKM2-KO mice. All data are presented as the mean ± SD. ***P*< 0.01. **(D)** The mRNA level of *SERPINE1* (normalized to that of *GAPDH*) in mammary tumors from control and PKM2-KO mice was analyzed by RT‒qPCR. All data are presented as the mean ± SD. **P*< 0.05.

Table S1. Mass spectrometry results (Top 30)


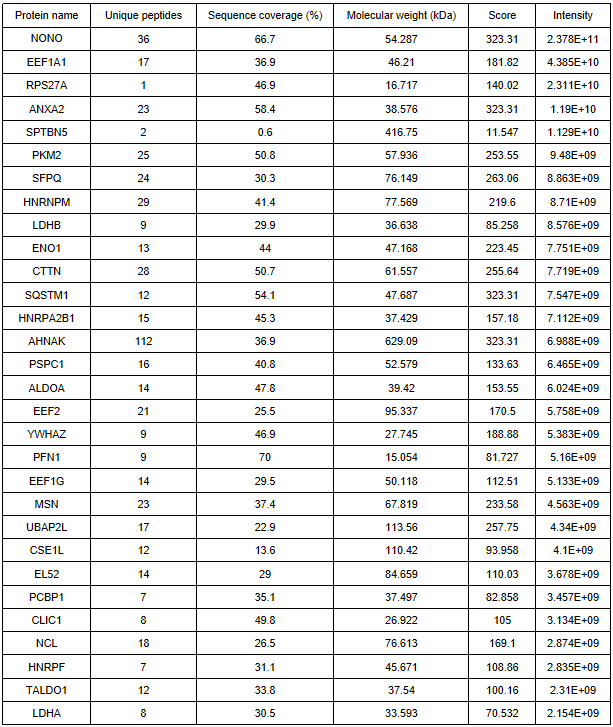


Table S2. Clinicopathologic characteristic of NONO expression in TNBC patients


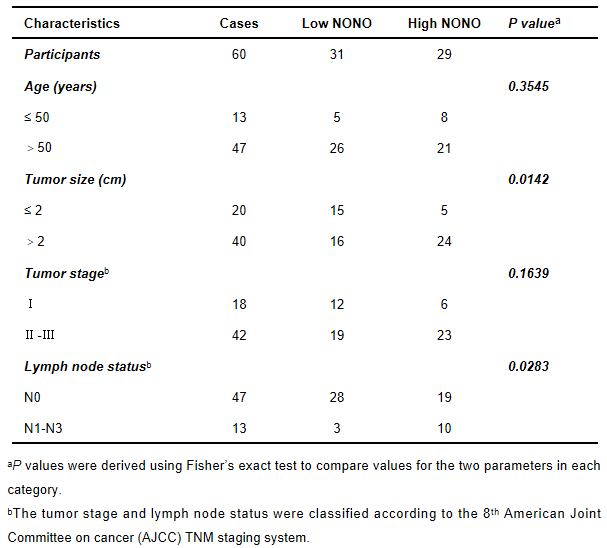


Table S3. Clinicopathologic characteristic of PKM2 expression in TNBC patients


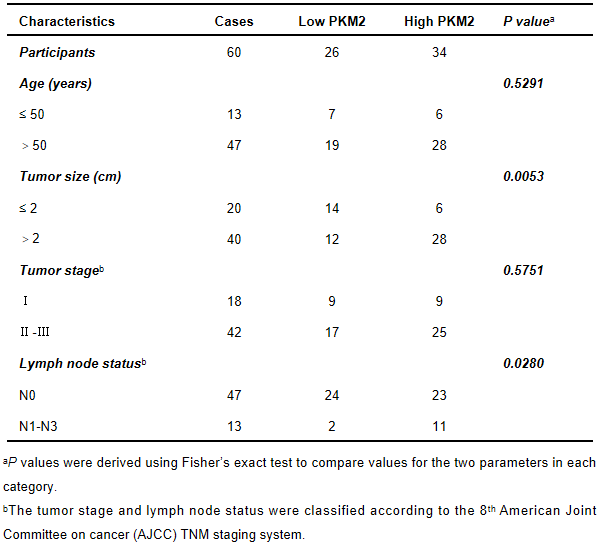


Table S4. List of antibodies


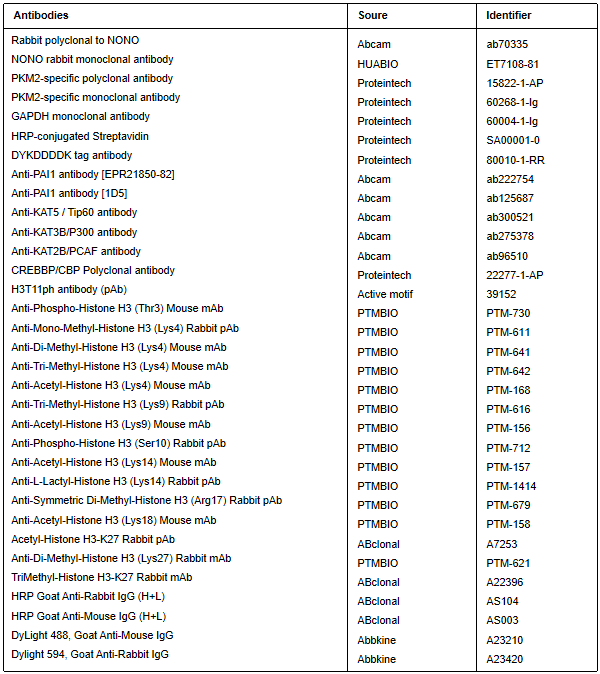


Table S5. Real-time PCR primer sequences


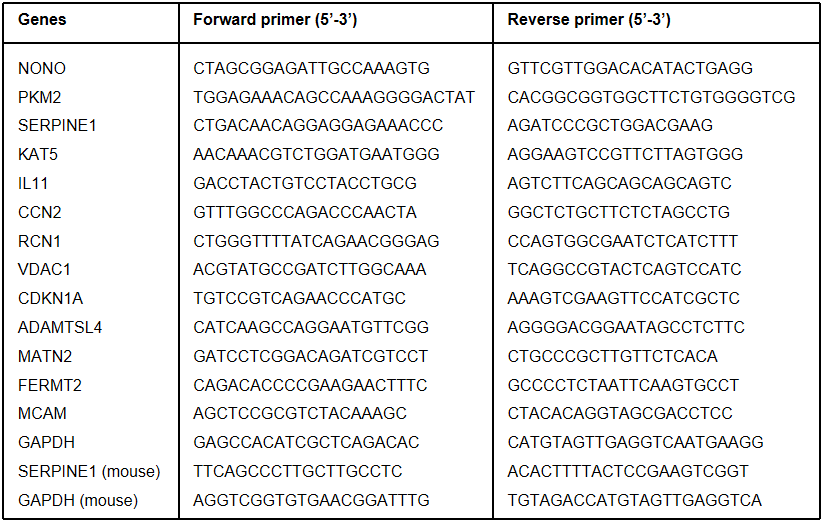


Table S6. ChIP-qPCR primer sequences


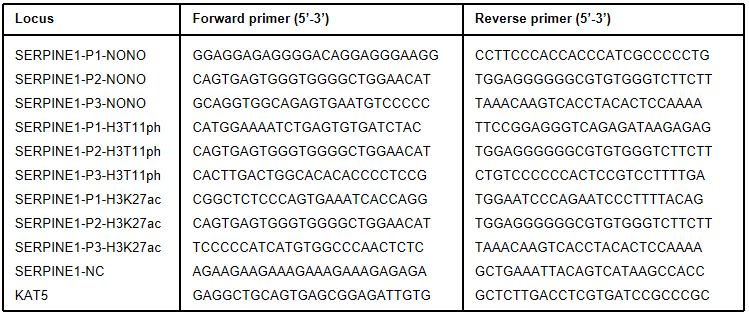


Table S7. PCR primer sequences used for genotyping of transgenic mice


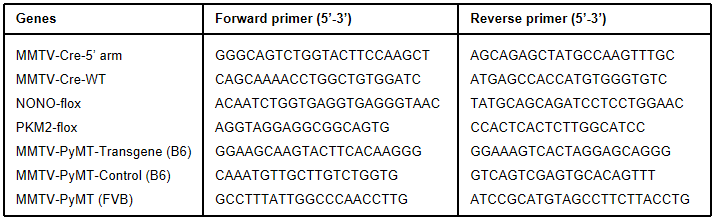

Supplement: Supplementary file 1 — Supplementary Material 1 [file 13046_2025_3343_MOESM1_ESM.docx]
